# Mechanistic basis for potent neutralization of Sin Nombre hantavirus by a human monoclonal antibody

**DOI:** 10.1038/s41564-023-01413-y

**Published:** 2023-06-15

**Authors:** Robert Stass, Taylor B. Engdahl, Nathaniel S. Chapman, Rachael M. Wolters, Laura S. Handal, Summer M. Diaz, James E. Crowe, Thomas A. Bowden

**Affiliations:** 1 Division of Structural Biology, Wellcome Centre for Human Genetics, University of Oxford, Oxford, UK; 2 Department of Pathology, Microbiology and Immunology, Vanderbilt University Medical Center, Nashville, TN USA; 3 Vanderbilt Vaccine Center, Vanderbilt University Medical Center, Nashville, TN USA; 4 Department of Pediatrics, Vanderbilt University Medical Center, Nashville, TN USA

**Keywords:** X-ray crystallography, Viral infection, Antibodies

## Abstract

Rodent-borne hantaviruses are prevalent worldwide and upon spillover to human populations, cause severe disease for which no specific treatment is available. A potent antibody response is key for recovery from hantavirus infection. Here we study a highly neutralizing human monoclonal antibody, termed SNV-42, which was derived from a memory B cell isolated from an individual with previous Sin Nombre virus (SNV) infection. Crystallographic analysis demonstrates that SNV-42 targets the Gn subcomponent of the tetrameric (Gn−Gc)_4_ glycoprotein assembly that is relevant for viral entry. Integration of our 1.8 Å structure with the (Gn−Gc)_4_ ultrastructure arrangement indicates that SNV-42 targets the membrane-distal region of the virus envelope. Comparison of the SNV-42 paratope encoding variable genes with inferred germline gene segments reveals high sequence conservation, suggesting that germline-encoded antibodies inhibit SNV. Furthermore, mechanistic assays reveal that SNV-42 interferes with both receptor recognition and fusion during host-cell entry. This work provides a molecular-level blueprint for understanding the human neutralizing antibody response to hantavirus infection.

## Abstract (web summary)

Structural analysis reveals how a potent human monoclonal antibody neutralizes Sin Nombre virus by binding the Gn–Gc heterodimer lattice.

## Main

Sin Nombre virus (SNV) is a rodent-borne virus that can cause severe respiratory failure and death upon spillover from their reservoir host into humans. In 1993, SNV was first identified as the aetiological agent of an outbreak of a mysterious, flu-like illness in the Four Corners region of the United States, representing the first case of hantaviruses detected in the Americas^1^. SNV is an enveloped, negative-sense RNA virus in the *Orthohantavirus* genus in the *Hantaviridae* family. The mysterious illness, characterized by respiratory failure with diffuse interstitial oedema, was later named Hantavirus Cardiopulmonary Syndrome, and thousands of cases have been reported in North and South America since the 1990s^2,3^. SNV causes a persistent infection in deer mice (*Peromyscus maniculatus*) and can be transmitted to humans through the aerosolization of rodent excreta. Person-to-person transmission has been suggested for Andes virus (ANDV), the predominant New World hantavirus (NWH) in South America^4^. There are few medical countermeasures for any hantavirus-related infection or disease. Therefore, NWHs represent a substantial epidemic threat.

The hantavirus integral membrane surface glycoproteins, designated Gn and Gc, form hetero-oligomeric complexes on the virion surface containing four copies of each glycoprotein. These complex spikes facilitate viral attachment and fusion with the host-cell membrane. X-ray crystallography studies have elucidated the structure of the ectodomain of Gn, the N-terminal Gn head region (termed Gn^H^), for NWHs including ANDV and Maporal virus (MAPV)^5^, and Old World species such as Hantaan virus (HTNV)^6^ and Puumala virus (PUUV)^7^. The Gn^H^ forms a β-sandwich fold characteristic of the attachment protein in multiple other class II viruses, including the E2 protein of alphaviruses and the Gn protein in phleboviruses^8–10^. Although the exact functions of Gn are not well understood, previous three-dimensional structural characterization of the glycoprotein complex demonstrated that Gn and Gc form a heterotetrameric complex, and a flexible loop (the ‘capping loop’) interfaces with the cd and bc loops of the membrane insertion region of Gc, probably preventing the premature induction of fusion by Gc^5^. Also, the surface-exposed region of Gn^H^ has high sequence variability across the hantavirus family, which may indicate its ability to interact with the NWH-specific receptor protocadherin-1 (PCDH-1)^11^. However, the location of the receptor-binding site on hantaviruses is unknown, and the immunodominant antigenic sites on Gn/Gc recognized by the human immune system are not defined^12^.

Previous studies determined the structural basis of neutralization by non-human monoclonal antibodies (mAbs) targeting Gc^13^ or Gn^14^. A bank vole mAb designated P4G2, isolated after experimental infection of a vole with PUUV, recognizes the prefusion form of Gc at the junction between the DI and DII domains^13^. Although Gc-specific mAbs targeting this site neutralize in pseudovirus assays, these mAbs typically neutralize authentic hantaviruses weakly, probably due to the cryptic nature of this epitope on Gc, as demonstrated by the higher-order structure of the glycoprotein spikes on the virion^5,15,16^. Potent hantavirus neutralizing antibodies (nAbs) typically target Gn^H^, and a rabbit nAb designated HTN-Gn1 targets a loop in domain A^14^. Additionally, low-resolution reconstructions of human PUUV nAbs in complex with the PUUV Gn^H^/Gc were determined using negative stain electron microscopy^15^. The antibodies targeted two sites: the first site is in the interface between Gn/Gc, engaging with the capping loop region on Gn, while the second site is in the DI/DII linker region in Gc (similar to the epitope recognized by P4G2). The molecular basis of human mAbs engagement with hantavirus glycoproteins has not yet been determined.

Previously, we isolated a panel of mAbs from individuals who were immune to SNV because of previous natural infection and demonstrated that one mAb, SNV-42, showed exceptionally potent neutralizing activity for SNV in vitro^16^. Here we determined the structure of SNV-42 in complex with SNV Gn^H^ at 1.8 Å resolution and determined the molecular basis for SNV-42-mediated neutralization of SNV. SNV-42 functions at two distinct steps in the viral entry pathway, blocking receptor binding and inhibiting viral fusion after virion attachment to host cells. Identifying the antigenic sites on the hantavirus glycoproteins recognized by neutralizing human antibodies is a critical part of understanding the natural human response to hantavirus infection and may inform rational structure-based design of therapeutic antibodies and vaccines.

## Results

### A germline-revertant form of SNV-42 neutralizes SNV

**Fig. 1:**
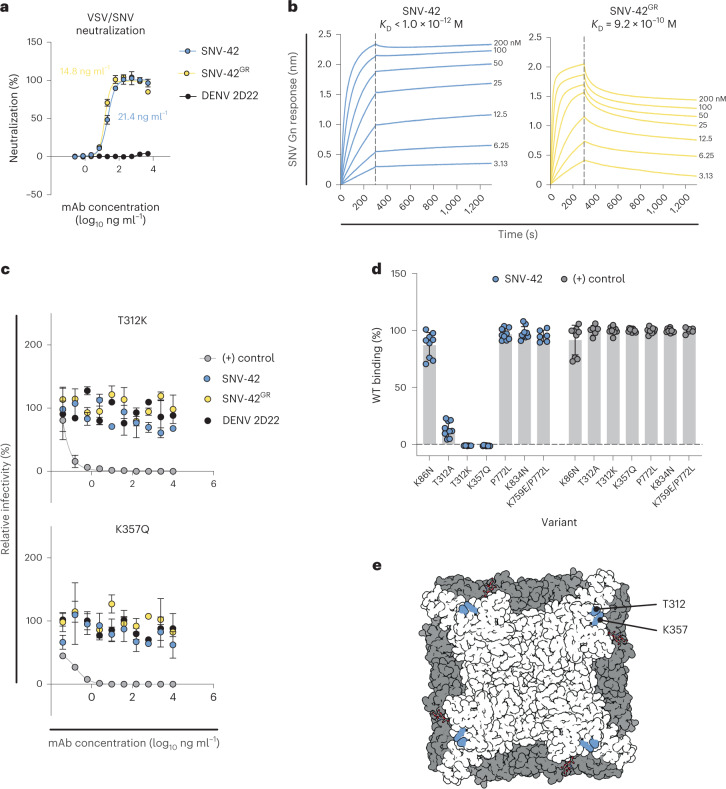
Viral variants capable of escaping neutralization. **a**, Representative neutralization curves of SNV-42, germline reverted (GR) SNV-42 and negative control DENV 2D22 to VSV/SNV determined through real-time cellular analysis using the Vero CCL-81 cell line. IC_50_ values were calculated on the basis of a nonlinear regression and error bars denote mean ± s.d. The assay was performed three independent times with similar results. **b**, Affinity measurements of SNV-42 and SNV-42^GR^ for binding to SNV Gn^H^ ectodomain, measured by bio-layer interferometry. Representative curves and *K*_D_ values are shown for SNV-42^GR^, while the *K*_D_ value for SNV-42 could not be determined because the *K*_off_ could not be measured. Dashed line indicates dissociation step at 300 s. **c**, Representative neutralization curves of SNV-42, SNV-42^GR^, positive control (oligoclonal mix of SNV-reactive antibodies) and DENV 2D22 to mutant VSV/SNV viruses. Error bars denote mean ± s.d. The assay was performed three independent times with similar results. **d**, SNV-42 binding in the presence of SNV M-segment mutant constructs determined by flow cytometry. The percent binding (% WT) of each mAb to the mutant constructs was compared to the WT SNV construct. An oligoclonal mix of SNV-reactive antibodies was included to control for expression of each mutant construct. The data are shown as mean ± s.d.; from left to right, *n* = 9, 9, 9, 9, 9, 9, 6, 9, 9, 9, 9, 9, 9 and 6 technical replicates. The assay was performed three to four independent times with similar results. **e**, Top view of escape mutants mapped to the ANDV Gn/Gc spike (PDB: 6ZJM). The blue residues designate escape mutants. Gn is shown in white and Gc is shown in grey. All numbering for SNV sequences was based on GenBank KF537002.1.

Previous sequence analysis determined that SNV-42, which is encoded by human antibody variable region gene segments IGHV3-48*03/IGLV1-40*01, is remarkably close in sequence to the germline-encoded sequence, with a 97 or 99% identity to the inferred heavy and light chain variable gene sequences, respectively^16^. To understand whether somatic mutations are necessary for potent neutralization activity, we aligned the SNV-42 coding sequence with the inferred germline gene segments and reverted all mutations in the antibody variable regions to the residue encoded by the inferred germline gene (Supplementary Fig. 1). We then performed a neutralization assay to compare the potency of SNV-42 and the germline-revertant (GR) form of that antibody (denoted as SNV-42^GR^). We did not detect a change in the IC_50_ value between SNV-42 (IC_50_ = 21.4 ng ml^−1^) and SNV-42^GR^ (IC_50_ = 14.8 ng ml^−1^) **(**Fig. 1a). Given that some of the residues in those regions are non-templated and thus cannot be reverted, we did not alter the junctional regions of SNV-42. These results indicate that many of the residues in the antibody paratope that are critical for SNV neutralization are encoded by *IGHV3-48/IGLV1-40* germline genes. We also measured the *K*_D_ values for the affinity matured and the germline reverted forms of SNV-42 to the recombinant SNV Gn head domain (Fig. 1b and Supplementary Table 1) using bio-layer interferometry (BLI). SNV-42 bound to Gn^H^ with sub-picomolar affinity, while SNV-42^GR^ demonstrated sub-nanomolar affinity (9.2 × 10^−10^ M). However, this difference in affinity does not appear to impact the neutralization potency.

### Critical residues for SNV-42 binding

Identifying potential escape mutants for antibodies is a crucial part of therapeutic development, and methods of immune evasion employed by hantaviruses are not well understood. To identify the critical binding residues involved in the recognition of SNV Gn by SNV-42, we used two different methods to identify escape mutants resistant to neutralization mediated by SNV-42. First, we implemented a high-throughput, single-passage neutralization escape mapping method using a real-time cellular analysis (RTCA) cell-impedance-based technology. We identified escape mutants in 32 of 88 replicates tested for escape, as manifested by cytopathic effect (CPE) in the presence of neutralizing concentrations of SNV-42 (Supplementary Fig. 2). We sequenced the gene encoding Gn in the virus in the supernatants in 6 wells. The neutralization-resistant viruses contained Gn gene mutations encoding K357Q or T312K alterations (Fig. 1c,e). To further identify escape mutants, we also selected a similar escape mutant (T312A) by serial passaging of a recombinant vesicular stomatitis virus (VSV/SNV) in increasing concentrations of SNV-42. We expressed recombinant forms of Gn with these mutations on the surface of cells and tested binding of SNV-42 to the mutant Gn constructs in flow cytometric binding assays. All three mutations completely ablated mAb binding, further supporting that these two residues are critical binding contacts (Fig. 1d). The binding of SNV-42 was not impacted by escape mutations selected for other SNV-neutralizing mAbs recognizing different antigenic sites (SNV-53 and SNV-24). Taken together, these two methods identified critical residues on SNV Gn that may be under pressure by some antibodies elicited in the human immune response to infection. However, SNV field strains with mutations at T312 or K357 have not been reported.

### Structure of SNV-42 in complex with SNV Gn protein

To understand the structural basis for SNV Gn recognition of SNV-42, a construct encoding the SNV Gn^H^ head domain (residues 21−377) was produced recombinantly, purified and complexed with the Fab component of SNV-42. The SNV Gn^H^−SNV-42 complex was subjected to size exclusion purification and crystallized, and the structure was determined to approximately 1.8 Å resolution.

**Fig. 2:**
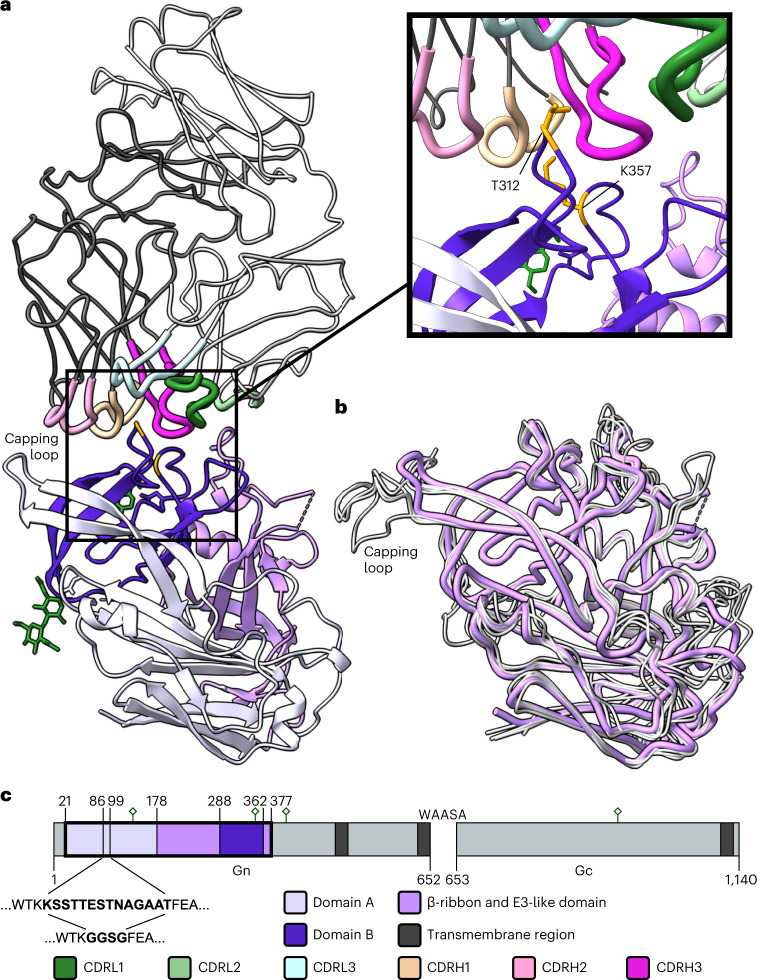
Crystal structure of Sin Nombre GnH in complex with Fab SNV-42. **a**, Structure of the Gn–Fab complex. The Fab is displayed with the backbone of the light and heavy chains coloured light grey or dark grey, respectively. The CDR loops are thicker and coloured according to the key in **c**. The Gn is displayed as a ribbon diagram with each of the three domains coloured according to the key in **c**. The two N-linked glycosylation sites are displayed in green and the location of the two previously described escape mutants (T312K and K357Q) are displayed in orange. Inset is a zoomed panel of the binding site with the side chains of the two escape mutant residues displayed. **b**, The backbone of the SNV Gn^H^ in pink overlaid on several previously reported Gn^H^ crystal structures from different hantavirus species in grey. These include Andes orthohantavirus (PDB ID 6Y5F), Maporal orthohantavirus (PDB ID 6Y62), Puumala orthohantavirus (PDB ID 5FXU) and Hantaan orthohantavirus (PDB ID 5OPG). Of note is the capping loop, indicated, which was replaced in SNV Gn^H^ with a much shorter GGSG linker to aid crystallogenesis. **c**, A domain schematic of the Sin Nombre glycoprotein precursor protein that is cleaved at the WAASA cleavage site to form Gn and Gc. The crystallized Gn^H^ region is outlined in bold and coloured according to domain. Transmembrane regions are displayed in dark grey and N-linked glycosylation sites displayed in green. The sequence of the capping loop between residues 86–99 is displayed alongside the shorter GGSG linker that has been used in its place for this experiment.

One complex of SNV Gn−SNV-42 was observed in the asymmetric unit of the unit cell (Fig. 2). The structure of SNV Gn^H^ has not been reported previously and consists of a compact fold formed of three domains: domains A and B and a β-ribbon domain (Fig. 2). Despite a relatively low level of sequence identity (ranging from 43 to 63%), the SNV Gn^H^ is very similar to previously characterized hantavirus Gn glycoproteins^5–7,14^, where the equivalent regions of MAPV Gn^H^, ANDV Gn^H^, PUUV Gn^H^ or HNTV Gn^H^ exhibit root-mean-square deviations of 0.7, 0.9, 1.0 or 1.0 Å over equivalent Cα residues, respectively (Fig. 2b). Regions of SNV Gn^H^ that exhibit the greatest level of structural deviation from other Gn^H^ structures are in solvent exposed loop regions, consistent with these areas of the molecule being naturally flexible or requiring stabilizing contacts from the higher-order (Gn−Gc)_4_ assembly.

Consistent with the epitope mapping analysis (Fig. 1d), SNV-42 binds to domain B and the E3-like domain of SNV Gn (Fig. 2a). The residues implicated in antibody escape identified above, T312 and K357, form key hydrogen bonding interactions with CDRH3 and CDRH1/3, respectively. These hydrogen bonding interactions appear to be perturbed when the mutations T312K and K357Q are modelled, and some rotomeric configurations of K312 may sterically interfere with the antibody, providing a structural rationale for antibody escape (Supplementary Fig. 4). The epitope comprises a large glycan-independent interface, which occludes ~800 Å^2^ of buried surface area. While all complementarity-determining regions (CDRs) contribute to the epitope, residues comprising the CDRH3 of SNV-42 form the bulk of the interaction through insertion of a 9-residue-long loop into a cleft formed on the SNV Gn surface (Supplementary Fig. 3). CDRH3 possesses a low number of sequence somatic mutations from the putative germline, with only a single amino acid change from the germline D-gene (IGHD5-12*01). This change is one of only five amino acid changes from the germline-encoded sequence present in the paratope region including CDRH1 (T36), CDRH1 (E38), CDRH2 (R57) and CDRH3 (T112) that were all originally encoded as serine residues, plus CDRL1 (Y38) that was originally encoded as aspartate. However, these mutations do not impact the neutralization potency of SNV-42 (Fig. 1a and Supplementary Fig. 1). Interestingly, none of these five paratope residues were observed to sterically hinder antigen recognition when modelled back to the germline-encoded sequence (Supplementary Fig. 5).

SNV-42 is highly specific to SNV and did not demonstrate reactivity to or neutralize any other hantaviruses tested previously^16^. Assessment of sequence conservation at the epitope provides a structural rationale for this observation, since only 12 of 20 residues in the SNV-42 epitope were conserved with ANDV and 8 of 20 with HTNV. Furthermore, among these non-conserved residues, there exist non-complementary side chains which would probably sterically preclude mAb recognition (Supplementary Fig. 6).

### SNV-42 targets the membrane-distal region of Gn−Gc

**Fig. 3:**
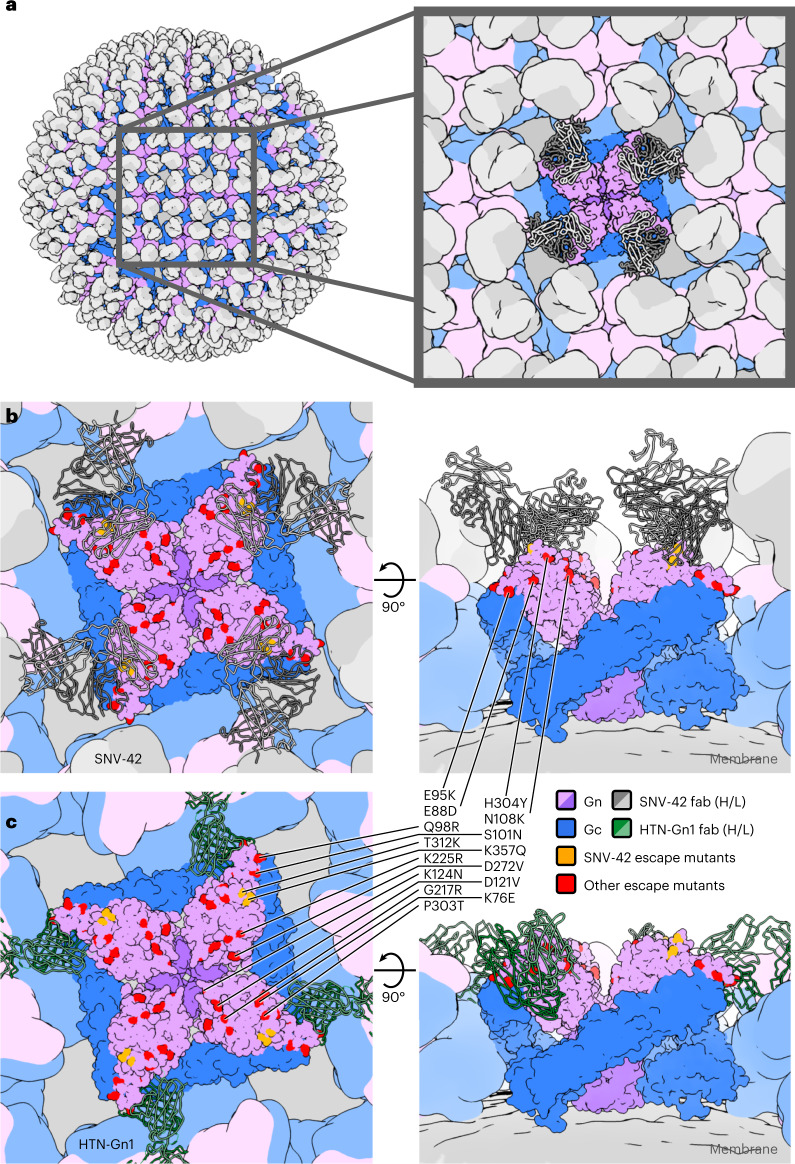
A model of the Sin Nombre glycoprotein lattice in complex with Fab SNV-42. **a**, An EM-derived model of a Sin Nombre virion decorated in Fab fragments of mAb SNV-42. The virion model is derived from previously reported cryoET data of Tula virus^5^. The Gc is coloured blue and the Gn coloured pink or purple for the head or stalk regions, respectively. The light or heavy chains of the Fab are coloured light or dark grey, respectively. The zoomed inset displays nine individual glycoprotein spikes with the central spike surface rendered at higher resolution. The Fab fragments bound to the central spike are displayed as a backbone trace. **b**, Top view (left) and side view (right) of the Sin Nombre glycoprotein spike bound to Fab fragments of SNV-42. This assembly model is based on the previously reported ANDV glycoprotein spike tetramer (PDB: 6ZJM). The location of two SNV-42 escape mutants (T312K and K357Q) are displayed in orange and the equivalent locations of other previously reported antibody escape mutants are displayed in red. The complete list of antibody escape mutants and the species they apply to are detailed in Supplementary Table 2. To enable visualization of all epitopes, two loops that are not resolved in this SNV Gn structure (residues 86–99 and 221–229) were replaced by their equivalents from a previously reported ANDV Gn structure (PDB: 6ZJM). **c**, The equivalent view of a hantavirus glycoprotein spike bound to Fab HTN-Gn1, a previously reported neutralizing antibody that binds to HNTV^14^.

Previous integrative cryo-electron tomography (cryoET) and X-ray crystallography analyses of ANDV, PUUV, HTNV and Tula virus (TULV) have revealed that the ultrastructure arrangement of the hantaviral (Gn−Gc)_4_ is well conserved and consists of a tetramer of Gn−Gc heterodimers. The Gn^H^ forms the most membrane-distal region of the spike and shields fusion loops located in domain II of the Gc^5,7,13^. To assess the location of the SNV-42 epitope in the context of the higher-order hantaviral Gn−Gc lattice, we overlayed the SNV Gn subcomponent of our complex onto a previously reported (Gn−Gc)_4_ assembly of ANDV (PDB: 6ZJM) (Fig. 3a,b). This analysis demonstrates that SNV-42 binds to the membrane-distal region of the lattice. While spatially distinct, the SNV-42 epitope is proximal to and slightly overlaps with the epitope of the weakly neutralizing mAb, HTN-Gn1^14^, the only other structurally characterized anti-Gn mAb (Fig. 3c). In contrast to HTN-Gn1, SNV-42 binds in an orientation that is relatively perpendicular to the membrane (Fig. 3b,c). We note that each of the SNV-42 epitopes on the (Gn−Gc)_4_ tetramer is mutually accessible for binding. Furthermore, unlike for HTN-Gn1, these sites are equally accessible in a cryo-electron microscopy (cryoEM)-derived model of the entire virus with the location of the (Gn−Gc)_4_ spikes mapped onto the virion surface (Fig. 3a).

Previous epitope mapping of a panel of human SNV Gn- and Gc-specific antibodies revealed a series of epitopes spanning across solvent-accessible surfaces of the higher-order (Gn−Gc)_4_ spike^15,16^. Integration of these data with putative epitopes predicted on the surface of other New and Old World hantaviruses indicates a broad distribution of epitopes across both the Gn and Gc glycoproteins (Fig. 3b). While immunodominant regions that are targeted during infection and immunization have yet to be identified, one such epicentre exists at the membrane-distal region of the Gn^H^ glycoprotein and co-localizes with our structurally elucidated SNV-42 epitope.

### Bivalent interactions drive neutralization

**Fig. 4:**
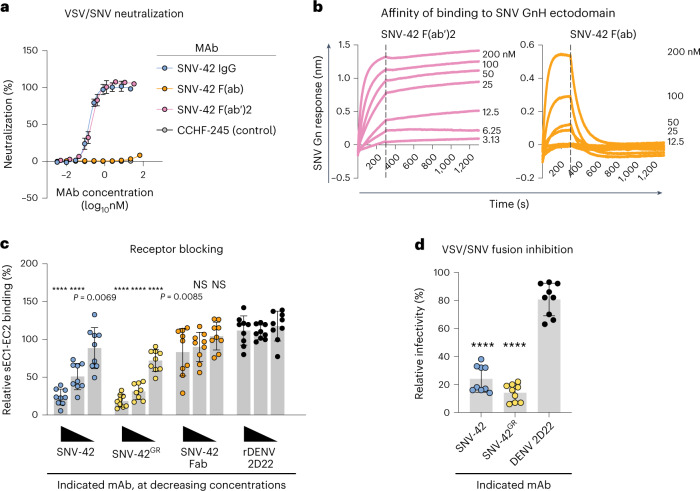
SNV-42 neutralization is dependent on bivalency and occurs by combination of receptor blocking and fusion inhibition. **a**, Representative neutralization curves of IgG1 and Fab forms of SNV-42 to VSV/SNV determined by RTCA using the Vero CCL-81 cell line. IC_50_ values were calculated on the basis of a nonlinear regression and error bars denote mean ± s.d. The assay was performed three independent times with similar results. **b**, Representative affinity curves of the F(ab′)2 and F(ab) form of SNV-42 for binding to SNV Gn^H^, measured by bio-layer interferometry. Representative curves and *K*_D_ values are shown for SNV-42 F(ab), while the *K*_D_ value for SNV-42 F(ab′)2 could not be determined because the *K*_off_ could not be measured. Dashed line indicates the dissociation step at 300 s. **c**, sEC1-EC2 blocking activity of neutralizing antibodies determined through a flow cytometric assay in which mAbs were added at saturating concentration before the addition of the soluble PCDH-1 domain labelled with Alexa Fluor 647 dye. High (50 µg ml^−1^), medium (10 µg ml^−1^) or low (0.5 µg ml^−1^) mAb concentrations were tested. Two-way analysis of variance (ANOVA) with Dunnett’s multiple comparisons, ****P < 0.0001; NS, not significant. The data are shown as mean ± s.d., *n* = 9 technical replicates. The assay was performed two independent times with similar results. **d**, FFWO assay testing VSV/SNV post-attachment antibody neutralization in permissive (pH 5.5) conditions at 10 µg ml^−1^. Vero CCL-81 cells were used and GFP expression was measured to determine relative infectivity. The data are shown as mean ± s.d. of technical replicates, *n* = 9. The assay was performed two independent times with similar results. One-way ANOVA with Dunnett’s multiple comparisons, ****P < 0.0001.

The role of bivalent interactions in the neutralization potency of hantavirus antibodies has yet to be described. To determine how the avidity effects impact the potency of SNV-42, we performed a neutralization assay comparing SNV-42 as a full-length IgG, Fab and F(ab′)2. The F(ab′)2 form was included to rule out any contributing steric effects of the fragment crystallizable (Fc) domain in neutralizing the virus. We saw no difference in the neutralizing activity between the full-length IgG form and the F(ab′)2 form; however, the Fab form of SNV-42 did not demonstrate detectable neutralizing activity for VSV/SNV **(**Fig. 4a**)**.

To further determine whether the lack of neutralizing activity was due to loss in avidity, we measured the *K*_D_ values of the Fab and F(ab′)2 forms of SNV-42 to SNV Gn^H^ using BLI (Fig. 4b). In concordance with the IgG form, the F(ab′)2 bound SNV Gn^H^ with a sub-picomolar avidity, while the Fab form demonstrated a fast off rate and low *K*_D_ value in comparison (4.08 × 10^−8^ M). Thus, the neutralization activity of SNV-42 requires bivalent binding to the (Gn−Gc)_4_ assembly.

To investigate the possibility that bivalent binding of SNV-42 disrupts fusogenic conformational changes to the Gn–Gc complex, we assessed the likelihood of SNV-42 to cross-link neighbouring epitopes on the (Gn−Gc)_4_ assembly (Supplementary Fig. 9). This analysis suggests that inter-spike, but not intra-spike, bivalent binding may be plausible.

### SNV-42 inhibits multiple steps during SNV entry

The hantavirus Gn protein probably plays several roles in facilitating the entry of hantavirus virions into host cells^17^. Gn forms a heterodimer with Gc and prevents the premature membrane insertion of the virus by covering the hydrophobic residues in the fusion loop^5^. Although the receptor-binding site is unknown, Gn is assumed to interact with attachment factors, including PCDH-1^17^. To understand how SNV-42 engages with Gn to neutralize SNV, we investigated two mechanisms of interfering with viral entry: receptor blocking and fusion inhibition. Previous work has shown that the first extracellular cadherin-repeat domain (EC1) of PCDH-1 interacts with SNV Gn/Gc^11^, so we employed a flow cytometry-based competition-binding assay to test whether SNV-42 could block the interaction of a soluble recombinant form of the EC domains (sEC1-EC2). We showed that SNV-42 and SNV-42^GR^ both block sEC1-EC2 binding to SNV Gn/Gc in a dose-dependent manner. However, we did not see complete blocking, even at the highest concentrations tested (50 µg ml^−1^; Fig. 4c). Notably, the Fab form of SNV-42 also did not block receptor binding, further suggesting that bivalent binding is required for receptor blocking and viral neutralization.

Although Gc is the canonical fusogenic protein, it is possible that targeting Gn may inhibit dynamic changes necessary to expose the fusion loop and promote viral entry^18–20^. We used a fusion from without (FFWO) assay to test fusion inhibition that can measure antibody-mediated neutralization post-attachment of the virion to the cell surface. SNV-42 and SNV-42^GR^ significantly reduced VSV/SNV infection but did not completely inhibit viral fusion even at saturating concentrations (Fig. 4d). Further, although it is uncertain whether the hantaviral Gn remains bound to the Gc throughout the host-cell entry process, superposition of the SNV Gn−SNV-42 complex onto the structure of ANDV Gn bound to the ‘near’ post-fusion state of ANDV Gc^15^ suggests that SNV-42 is also capable of recognizing a post-fusion state of the Gn–Gc complex (Supplementary Fig. 10). While the precise transitions undertaken by the Gn−Gc assembly are not well understood, it is plausible that bivalent binding of SNV-42 to the (Gn−Gc)_4_ lattice interferes with the structural transitions required for entry and fusion. As SNV-42 does not mediate complete receptor blocking or neutralization post-attachment at high concentrations, the findings suggest that both mechanisms probably contribute together to cause the exceptional potency of the antibody.

## Discussion

A robust humoral immune response is critical for surviving hantavirus infection^21^, and passive transfer of human antibodies has shown therapeutic potential^16,22^. Nevertheless, our previous knowledge of the molecular and structural basis for hantavirus neutralization by human antibodies is limited. Epitopes of human mAbs isolated from PUUV-convalescent donors were recently described to target Gc and the Gn−Gc interface^15^, but Gn-specific epitopes were not described. Here we provide the structural basis of potent neutralization of SNV by a near-germline-encoded mAb, SNV-42, isolated from a memory B cell of a convalescent SNV donor^16^. We demonstrate that SNV-42 targets an epitope in domain B on Gn^H^ and probably interferes with multiple steps of the viral entry pathway.

Previous structural studies and epitope mapping analyses identified several regions on the hantavirus (Gn−Gc)_4_ spike surface that can be targeted by neutralizing antibodies^13,14,23–25^. Gn^H^ is predominantly solvent-accessible and highly variable across species, probably indicating its immunodominance in eliciting neutralizing antibodies during infection^5^. Although topologically different, the hantavirus Gn^H^ exhibits a configuration similar to related bunyaviruses, including severe fever with thrombocytopenia syndrome virus (SFTSV, also known as Dabie bandavirus) and Rift Valley fever virus (RVFV)^7,10,26,27^. MAbs targeting Gn of RVFV and SFTSV that were isolated from convalescent survivors also had low frequencies of somatic mutations but still demonstrated potently neutralizing activity in vitro and protection^26,28^. Similar to SNV-42, these mAbs also targeted domain B on Gn and did not cross-react with the Gn of related phleboviruses. Protective human and animal mAbs isolated from RVFV vaccines and immunization with recombinantly derived RVFV Gn, respectively, also target domain B^29,30^.

Positioned at the membrane-distal region of the heterotetrameric spike, the Gn head domain is probably under appreciable humoral immune pressure^8^. Residues K357 and T312 could represent hotspots for hantaviral immune escape, but surveillance of SNV isolates is limited. Thus, it is unclear whether these mutations can be tolerated by authentic viruses, or how rapid viral evolution occurs in nature. Although Gc is more shielded from immune pressure than Gn and thus more conserved, the cryptic nature of Gc epitopes probably presents challenges for eliciting completely neutralizing and effective immune responses. Mitigating complete antibody escape and developing a broader therapeutic might be accomplished by delivering SNV-42 in combination with recently described broadly neutralizing mAbs targeting the Gn/Gc interface^15,16^.

We also demonstrate that a near-germline antibody has remarkable affinity and neutralization potency for Sin Nombre virus, and the observed somatic mutations do not seem to impact the potency of the antibody substantially. Given the high sequence conservation of paratope residues in SNV-42 compared with the inferred germline gene-encoded antibody, it seems likely that there is a relatively low hurdle for affinity maturation to generate protective mAbs against SNV Gn. Marked numbers of somatic mutations are necessary for developing many broadly neutralizing antibodies recognizing HIV envelope protein, while the reactivity of some influenza anti-stem-region antibodies develops rapidly through limited mutations in the *IGHV1-69* heavy chain variable gene^31^. Additionally, multiple classes of antibodies targeting the SARS-CoV-2 receptor binding domain, including potently neutralizing mAbs, have germline-encoded binding motifs^32–34^. The finding that many antibody residues that contact virus protein are germline-encoded has implications for eliciting an effective antibody response through vaccination. For instance, targeting stimulation of germline-encoded predecessors of mature bnAbs has been a central strategy in structure-based vaccine development for many virus families including *Coronaviridae*, *Orthomyxoviridae*, *Paramyxoviridae*, *Pneumoviridae* and *Filoviridae*^35^. However, it is notable that the CDRH3 loop contributes to the bulk of the interactions with Gn, hence it is possible that the non-templated junctional region may be critical to the potency of SNV-42 and this recombination event may not be replicated in other individuals. Future work should employ deep sequencing of the B cell repertoire elicited during hantavirus infection to untangle the humoral response to hantaviruses.

PCDH-1 has been identified as a candidate receptor for New World hantaviruses; however, the molecular basis for the interactions of Gn/Gc with PCDH-1 is currently unknown and may require a quaternary (Gn–Gc)_4_ structure for binding^11,17^. Here we show that SNV-42 partially blocks PCDH-1 receptor binding in a valency-dependent manner. When combined with mechanistic and structural analyses, these data suggest that SNV-42 disrupts recognition of sEC1-EC2, but that the SNV-42 epitope does not necessarily overlap with that of the PCDH-1 binding site. Our findings also show that SNV-42 can mediate neutralizing activity after the virus particle attaches to cells. Despite targeting a site distant from the Gn/Gc interface, SNV-42 could possibly disrupt dynamic changes to uncap the fusion loop on Gc and prevent fusogenic activity after attachment^18^. Furthermore, the lower neutralization potency of the Fab form of SNV-42, compared with the IgG1 and F(ab′)2 forms, indicates that the bivalent structure of the mAb probably plays a role in neutralization. These data also indicate that the Fc portion of the antibody is not required for neutralization and high-affinity bivalent interactions probably drive potent neutralization by this antibody.

Since the first isolation and description of SNV in the Four Corners region of the United States, there have been over 800 cases reported in the United States, including an outbreak of 10 cases linked to a campsite in Yosemite, killing three people^36^. Albeit rare, SNV has notable epidemic potential through natural spillover events or intentional bioterrorism threats^3,37^. The closely related New World species, ANDV, also causes serious outbreaks in South America, including a large ‘super-spreader’ event in Argentina, where person-to-person transmission was suggested to occur^4,38^. Through characterization of a potently neutralizing epitope on Gn/Gc elicited during natural infection, this study provides a molecular-level blueprint for the rational development of anti-hantavirus mAbs with therapeutic potential.

## Methods

### Cell lines

Expi293F cells (human, female origin) were cultured in suspension at 37 °C in 8% CO_2_ with shaking at 125 r.p.m. in Freestyle F17 Expression Medium (Gibco) supplemented with 10% Pluronic F-68 and 200 mM of l-glutamine. ExpiCHO cells (hamster, female origin) were cultured in suspension at 37 °C in 8% CO_2_ with shaking at 125 r.p.m. in ExpiCHO Expression Medium (Thermo Fisher). Vero CCL-81 cells were cultured in Dulbecco’s modified Eagle medium (DMEM; Thermo Fisher) supplemented with 10% fetal bovine serum (FBS) at 37 °C in 8% CO_2_. All cell lines were tested monthly for mycoplasma and all samples were negative.

### Viruses

Replication-competent, recombinant VSV strains expressing the SNV glycoproteins in place of the VSV-G protein were kindly provided by K. Chandran and propagated as previously described^11^. Briefly, Vero CCL-81 cells were grown to ~70% confluency and inoculated with ~1 × 10^6^ plaque forming units of VSV/SNV in DMEM supplemented with 2% FBS and incubated at 32 °C for 3 d (or until ~90% of cells were green fluorescent protein (GFP)+). Supernatant was collected and clarified through a 0.2 μm PES filter and aliquoted and stored at −80 °C.

### Recombinant antibody expression and purification

ExpiCHO cells were transiently transfected using the ExpiCHO expression system (Gibco) with plasmids encoding human IgG1 or Fab complementary DNAs. The supernatant was collected from ExpiCHO cultures and filtered. Affinity purification of IgG1 or Fab from ExpiCHO cell supernatant was performed on HiTrap MabSelectSure columns (Cytiva) or CaptureSelectTM CH1-XL columns (Thermo Fisher) using an ÄKTA pure protein purification system (Cytiva).

### Recombinant production and purification of SNV Gn^H^

A construct encoding the N-terminal ectodomain region (residues 21−377) of SNV Gn (Genbank accession number AFV71282.1) was cloned into the pHLsec expression vector^39^. To facilitate expression and crystallization, a GGSG linker was inserted to replace residues in the capping loop region (residues 86−99) of the protein. SNV Gn^H^ was transiently expressed in HEK 293T cells in the presence of the alpha-mannosidase inhibitor, kifunensine^40^, or in Expi293F cells. Cell supernatant containing SNV Gn was diafiltrated into a buffer containing 10 mM Tris (pH 8.0) and 150 mM NaCl and purified by immobilized metal affinity chromatography using a 5 ml HisTrap fast-flow column, followed by size exclusion chromatography with a Superdex 200 increase 10/300 column into a buffer containing 10 mM Tris (pH 8.0) and 150 mM NaCl.

### X-ray crystallography of SNV Gn^H^ in complex with SNV-42

Purified SNV-42 Fab and deglycosylated SNV Gn^H^ were complexed in a 1.2:1 molar ratio before purification by size exclusion chromatography into a buffer containing 10 mM Tris (pH 8.0) and 150 mM NaCl. Purified complex was concentrated to 12.7 mg ml^−1^ and crystallized at room temperature using the vapour diffusion method^41^ in a precipitant containing 0.2 M tri-ammonium citrate (pH 7.0) and 20% w/v polyethylene glycol 3350. Crystals were cryoprotected in the precipitant containing 10% glycerol before flash freezing in liquid nitrogen.

Crystallographic data were collected at beamline i04, Diamond Light Source, with a Dectris Eiger2 XE 16M detector. Diffraction data were processed with XIA2^42^ and the structure was solved with PHASER^43^ using the structures of an HTNV Gn N-terminal ectodomain fragment (PDB ID 5OPG)^6^ and a human Fab (PDB ID 5UR8)^44^ as search models. The structure was iteratively built in COOT^45^ and refined in Phenix^46^ using TLS parameterization. MolProbity was used to assess model quality^47^. Data collection and refinement statistics are presented in Supplementary Table 3 and representative views of the electron density are displayed in Supplementary Fig. 12.

### Structural representations

ChimeraX^48^ was used to generate the structural models shown in the figures.

### BLI

For kinetic assays with BLI, Octet Red96 (Pall FortéBio) was used. After hydration of tips in kinetics buffer for 15 min, a 60 s baseline step was done. Recombinant histidine-tagged N-terminal ectodomain region of SNV Gn was immobilized to HIS1K biosensor tips (FortéBio) at 10 μg ml^−1^ in kinetics buffer (FortéBio) and loaded for 200 s. After a 30 s baseline step, antibody (SNV-42, SNV-42^GR^, F(ab′)2, F(ab) or rDENV-2D22) was allowed to associate with SNV Gn^H^ for 300 s in a serial dilution scheme starting at 200 nM at a 1:2 dilution. Following association, a 1,000 s dissociation step was performed in kinetics buffer. Data extrapolation of the equilibrium dissociation constant values was done with curve-fitting on the Analysis HT 12.2.0.2 software. Reference wells for the dissociation of antigen from the biosensor tips were used to subtract background from the association and dissociation steps. For the curve fits, a local fitting using a 1:1 model with Savitzky–Golay filtering was performed.

### RTCA neutralization

A neutralization assay using RTCA to detect virus-induced CPE was used as described previously^49^. Briefly, Vero cells were plated at 18,000 cells per well in a 96-well E-plate (Agilent) and allowed to attach to the well for 16–20 h at 37 °C. VSV/SNV (~1,200 IU per well) were mixed with mAb in duplicate in a volume of 100 µl using DMEM (supplemented with 2% low IgG FBS) and incubated for 1 h at 37 °C in 5% CO_2._ After cells reached confluency, the virus and mAb mixtures were added to the wells. Cellular impedance was measured every 15 min for 80 h total using a microelectronic biosensor system for cell-based assays (xCELLigence System, ACEA Biosciences) and data were analysed using the RTCA software (v2.1.0, Agilent/ACEA Biosciences). Percent neutralization was calculated by dividing the cellular impedance in each well by the average cellular impedance in wells containing medium only (no virus).

### RTCA antibody escape mutant virus generation

A modified protocol using the RTCA method described above generated escape mutant viruses after a single passage^49–51^. SNV-42 (20 µg ml^−1^) was incubated with VSV/SNV (~12,000 IU per well) for 1 h at 37 °C and then added to cells. Virus-only (no mAb) and medium-only (no virus) wells were included as controls. Escape mutant viruses were identified by a drop in cellular impedance over 96 h. Supernatants from wells containing neutralization-resistant viruses were expanded to 12-well plates in 20 µg ml^−1^ of SNV-42 to confirm escape-resistant phenotype and the presence of another SNV-neutralizing mAb, SNV-24, as a control. Supernatants were filtered and stored at −80 °C.

### VSV/SNV serial passaging

VSV/SNV (multiplicity of infection of ~1) was incubated with 100 ng ml^−1^ of SNV-42 for 1 h at 37 °C and added to Vero cells in a 12-well plate. Cells were monitored for GFP expression to confirm virus propagation. After ~75% GFP expression/CPE was observed, 200 µl of supernatant containing the selected virus was passaged to a new cell monolayer culture in increasing concentrations of SNV-42. After four passages, the variants were grown in the presence of 10 µg ml^−1^ of SNV-42 or SNV-24 to confirm escape from SNV-42 neutralization. Supernatants were filtered and stored at −80 °C.

### Sequence analysis of escape mutant viruses

Viral RNA from neutralization-resistant viruses was then isolated using a QIAamp Viral RNA extraction kit (QIAGEN). The SNV M gene cDNA was amplified with a SuperScript IV One-Step RT-PCR kit (Thermo Fisher) using primers flanking the M gene. The resulting amplicon (~3,500 bp) was purified using SPRI magnetic beads (Beckman Coulter) and sequenced by the Sanger sequence technique using primers giving forward and reverse reads of the entire M segment. The full-length M gene segments for the original VSV/SNV stock used were also confirmed by Sanger sequencing. Each read was aligned to the VSV/SNV genome using Geneious Prime (v2020.1.2) to identify mutations.

### Mutagenesis of SNV M-segment gene and binding analysis

pWRG/SN-M(opt)^52^ was mutagenized using the Q5 Site-Directed Mutagenesis kit (NEB, E0554S). Primers were designed using the NEBaseChanger tool (https://nebasechanger.neb.com/) and PCR reactions were performed according to manufacturer instructions. All mutants were sequenced and confirmed using Sanger DNA sequencing. Microscale transfections in 96-well blocks (Nest, 503162) of Expi293F cell cultures were performed using the Gibco ExpiFectamine 293 transfection kit (Thermo Fisher, A14525). Sequence-confirmed plasmid DNA was diluted in OptiMEM I serum-free medium, incubated for 20 min with ExpiFectamine 293 reagent (Gibco) and then added to Expi293F cell cultures. Cells were incubated with shaking at 1,000–1,500 r.p.m. inside a humidified 37 °C tissue culture incubator in 8% CO_2_ and collected at 48 h post-transfection. To determine whether mutations impacted SNV-42 binding, a flow cytometric assay was used as previously described^16^. Transfected Expi293F were plated into 96-well V-bottom plates at 50,000 cells per well in FACS buffer (2% ultra-low IgG FBS, 1 mM EDTA, D-PBS). SNV-42 or an irrelevant mAb negative control, rDENV-2D22, was diluted to 1 µg ml^−1^ in FACS buffer, incubated for 1 h at 4 °C, washed twice with FACS buffer, detected with goat anti-human IgG antibodies conjugated to phycoerythrin (1:1,000, Southern Biotech, 2040-09) and then stained with DAPI to exclude dead cells. Fluorescence was measured with an iQue Screener Plus flow cytometer (Intellicyt) and quantified using the manufacturer’s ForeCyt software. Percent wild-type (WT) values were generated by dividing the binding of the mutant by that of the unmutated construct. An oligoclonal mix of SNV-reactive Gn/Gc mAbs was used as a positive control.

### Receptor blocking assay

A receptor blocking assay employing the soluble domains of PCDH-1 was performed as previously described^16^. A construct of soluble extracellular cadherin-repeat 1 and 2 (sEC1-EC2, GenBank: NM_002587, residues 1–284) was synthesized and cloned into the pCMV mammalian cell expression vector by Twist Biosciences with a C-terminal GSG linker and decahistidine tag^11^. sEC1-EC2 was expressed in ExpiCHO cells, purified by affinity chromatography and then labelled with Alexa Fluor 647 (Thermo Fisher). Expi293F cells expressing the SNV Gn/Gc, as described previously^16^, were incubated with 50 µg ml^−1^, 10 µg ml^−1^ or 0.5 µg ml^−1^ of each mAb for 30 min at 4 °C. Alexa Fluor 647-labelled sEC1-EC2 was added directly to the cell suspension and mAb at a final concentration of 5 µg ml^−1^ and incubated for an additional 30 min at 4 °C. Cells then were washed and stained with 0.5 µg ml^−1^ of 40,6-diamidino-2-phenyl-indole (DAPI), and staining was measured with an iQue Screener Plus flow cytometer (Intellicyt). Percent positive cells were gated on the basis of sEC1-EC2 reactivity to mock-transfected cells to control non-specific binding (Supplementary Fig. 8). Binding in the presence of antibody was divided by the maximal binding signal of labelled sEC1-EC2 alone to determine the % receptor blocking.

### FFWO assay

Antibody-mediated fusion inhibition was measured using an assay previously described for other bunyaviruses^29^. Vero cells were plated at 18,000 cells per well in poly-d-lysine-coated 96-well plates and incubated at 37 °C for 16 h. Cells were washed with binding buffer (RPMI 1640, 0.2% BSA, 10 mM HEPES (pH 7.4) and 20 mM NH_4_Cl) and incubated at 4 °C for 15 min. VSV/SNV was diluted to a multiplicity of infection of ~2 in binding buffer, added to cells for 45 min at 4 °C and then washed to remove unbound viral particles. MAbs were diluted to 10 µg ml^−1^ in DMEM (2% FBS) and added to cells for 30 min at 4 °C. Fusion buffer (RPMI 1640, 0.2% BSA, 10 mM HEPES and 30 mM succinic acid at pH 5.5) was added to cells for 2 min at 37 °C. Control wells were incubated with RPMI 1640 (supplemented with 0.2% BSA and 10 mM HEPES (pH 7.4)) for 2 min at 37 °C. Cells were washed and incubated at 37 °C in DMEM (5% FBS, 10 mM HEPES and 20 mM NH_4_Cl (pH 7.4)). After 24 h, the medium was removed, the cells were imaged on a CTL ImmunoSpot S6 Analyzer (CTL) and GFP-positive cells were counted in each well using the Immunospot FluoroX software (v7.0.18.1). Relative infectivity (%) was calculated by dividing the number of GFP-positive cells in the mAb-treated wells by the average number of GFP-positive cells in the virus-only control wells (no mAb).

### Quantification and statistical analysis

Statistical analysis for each experiment is described in Methods and/or in the figure legends. All statistical analysis was done using Prism v8 (GraphPad).

### Reporting summary

Further information on research design is available in the Nature Portfolio Reporting Summary linked to this article.

## Supplementary information

Supplementary Information Supplementary Figs. 1–12 and Tables 1–3.

Reporting Summary

## Data Availability

Atomic coordinates and structure factors of the SNV Gn^H^ Fab SNV-42 complex have been deposited in the PDB (accession code PDB ID 8AHN). Materials used in this study will be made available but may require execution of a Materials Transfer Agreement. Source data are provided on Mendeley Data at https://data.mendeley.com/datasets/8fs7tgs9fs. The following sequences were used to design constructs for protein expression: SNV M segment, Genbank: AFV71282.1 and sEC1-EC2, GenBank: NM_002587. Two atomic models used for phasing of X-ray data were PDB ID 5OPG and PDB ID 5UR8. Additional atomic models used for data visualization were ANDV Gn/Gc spike (PDB: 6ZJM), Andes orthohantavirus Gn (PDB ID 6Y5F), Maporal orthohantavirus Gn (PDB ID 6Y62), Puumala orthohantavirus Gn (PDB ID 5FXU) and Hantaan orthohantavirus (PDB ID 5OPG).

## References

[1] Nichol ST (1993). Genetic identification of a hantavirus associated with an outbreak of acute respiratory illness. Science.

[2] Jonsson CB, Figueiredo LT, Vapalahti O (2010). A global perspective on hantavirus ecology, epidemiology, and disease. Clin. Microbiol. Rev..

[3] Hjelle B, Torres-Pérez F (2010). Hantaviruses in the Americas and their role as emerging pathogens. Viruses.

[4] Martinez VP (2005). Person-to-person transmission of Andes virus. Emerg. Infect. Dis..

[5] Serris A (2020). The hantavirus surface glycoprotein lattice and its fusion control mechanism. Cell.

[6] Rissanen I (2017). Structural transitions of the conserved and metastable hantaviral glycoprotein envelope. J. Virol..

[7] Li S (2016). A molecular-level account of the antigenic hantaviral surface. Cell Rep..

[8] Guardado-Calvo P, Rey FA (2021). The surface glycoproteins of hantaviruses. Curr. Opin. Virol..

[9] Guardado-Calvo P, Rey FA (2021). The viral class II membrane fusion machinery: divergent evolution from an ancestral heterodimer. Viruses.

[10] Hulswit RJG, Paesen GC, Bowden TA, Shi X (2021). Recent advances in bunyavirus glycoprotein research: precursor processing, receptor binding and structure. Viruses.

[11] Jangra RK (2018). Protocadherin-1 is essential for cell entry by New World hantaviruses. Nature.

[12] Engdahl TB, Crowe JE (2020). Humoral immunity to hantavirus infection. mSphere.

[13] Rissanen I (2020). Molecular rationale for antibody-mediated targeting of the hantavirus fusion glycoprotein. eLife.

[14] Rissanen I (2021). Structural basis for a neutralizing antibody response elicited by a recombinant Hantaan virus Gn immunogen. mBio.

[15] Mittler E (2022). Human antibody recognizing a quaternary epitope in the Puumala virus glycoprotein provides broad protection against orthohantaviruses. Sci. Transl. Med..

[16] Engdahl TB (2021). Broad and potently neutralizing monoclonal antibodies isolated from human survivors of New World hantavirus infection. Cell Rep..

[17] Mittler E (2019). Hantavirus entry: perspectives and recent advances. Adv. Virus Res.

[18] Bignon EA, Albornoz A, Guardado-Calvo P, Rey FA, Tischler ND (2019). Molecular organization and dynamics of the fusion protein Gc at the hantavirus surface. eLife.

[19] Guardado-Calvo P (2016). Mechanistic insight into bunyavirus-induced membrane fusion from structure-function analyses of the hantavirus envelope glycoprotein Gc. PLoS Pathog..

[20] Willensky S (2016). Crystal structure of glycoprotein C from a hantavirus in the post-fusion conformation. PLoS Pathog..

[21] Bharadwaj M, Nofchissey R, Goade D, Koster F, Hjelle B (2000). Humoral immune responses in the hantavirus cardiopulmonary syndrome. J. Infect. Dis..

[22] Garrido JL (2018). Two recombinant human monoclonal antibodies that protect against lethal Andes hantavirus infection in vivo. Sci. Transl. Med..

[23] Duehr J (2020). Neutralizing monoclonal antibodies against the Gn and the Gc of the Andes virus glycoprotein spike complex protect from virus challenge in a preclinical hamster model. mBio.

[24] Wang M, Pennock DG, Spik KW, Schmaljohn CS (1993). Epitope mapping studies with neutralizing and non-neutralizing monoclonal antibodies to the G1 and G2 envelope glycoproteins of Hantaan virus. Virology.

[25] Kikuchi M (1998). Characterization of neutralizing monoclonal antibody escape mutants of Hantaan virus 76118. Arch. Virol..

[26] Wu Y (2017). Structures of phlebovirus glycoprotein Gn and identification of a neutralizing antibody epitope. Proc. Natl Acad. Sci. USA.

[27] Halldorsson S (2018). Shielding and activation of a viral membrane fusion protein. Nat. Commun..

[28] Wang Q (2019). Neutralization mechanism of human monoclonal antibodies against Rift Valley fever virus. Nat. Microbiol..

[29] Chapman NS (2021). Potent neutralization of Rift Valley fever virus by human monoclonal antibodies through fusion inhibition. Proc. Natl Acad. Sci. USA.

[30] Allen ER (2018). A protective monoclonal antibody targets a site of vulnerability on the surface of Rift Valley fever virus. Cell Rep..

[31] Pappas L (2014). Rapid development of broadly influenza neutralizing antibodies through redundant mutations. Nature.

[32] Yuan M (2020). Structural basis of a shared antibody response to SARS-CoV-2. Science.

[33] Dong J (2021). Genetic and structural basis for SARS-CoV-2 variant neutralization by a two-antibody cocktail. Nat. Microbiol.

[34] Robbiani DF (2020). Convergent antibody responses to SARS-CoV-2 in convalescent individuals. Nature.

[35] Byrne PO, McLellan JS (2022). Principles and practical applications of structure-based vaccine design. Curr. Opin. Immunol..

[36] Núñez JJ (2014). Hantavirus infections among overnight visitors to Yosemite National Park, California, USA, 2012. Emerg. Infect. Dis..

[37] D’Souza MH, Patel TR (2020). Biodefense implications of New-World hantaviruses. Front. Bioeng. Biotechnol..

[38] Martínez VP (2020). ‘Super-spreaders’ and person-to-person transmission of Andes virus in Argentina. N. Engl. J. Med..

[39] Aricescu AR, Lu W, Jones EY (2006). A time- and cost-efficient system for high-level protein production in mammalian cells. Acta Crystallogr. D.

[40] Chang VT (2007). Glycoprotein structural genomics: solving the glycosylation problem. Structure.

[41] Walter TS (2005). A procedure for setting up high-throughput nanolitre crystallization experiments. Crystallization workflow for initial screening, automated storage, imaging and optimization. Acta Crystallogr. D.

[42] Winter G (2010). xia2: an expert system for macromolecular crystallography data reduction. J. Appl. Crystallogr..

[43] McCoy AJ (2007). Phaser crystallographic software. J. Appl. Crystallogr..

[44] Lopez-Sagaseta J (2018). Crystal structure reveals vaccine elicited bactericidal human antibody targeting a conserved epitope on meningococcal fHbp. Nat. Commun..

[45] Emsley P, Cowtan K (2004). Coot: model-building tools for molecular graphics. Acta Crystallogr. D.

[46] Adams PD (2002). PHENIX: building new software for automated crystallographic structure determination. Acta Crystallogr. D.

[47] Chen VB (2010). MolProbity: all-atom structure validation for macromolecular crystallography. Acta Crystallogr. D.

[48] Pettersen EF (2021). UCSF ChimeraX: structure visualization for researchers, educators, and developers. Protein Sci..

[49] Gilchuk P (2021). Pan-ebolavirus protective therapy by two multifunctional human antibodies. Cell.

[50] Greaney AJ (2021). Complete mapping of mutations to the SARS-CoV-2 spike receptor-binding domain that escape antibody recognition. Cell Host Microbe.

[51] Suryadevara N (2022). Real-time cell analysis: a high-throughput approach for testing SARS-CoV-2 antibody neutralization and escape. STAR Protoc..

[52] Hooper JW, Josleyn M, Ballantyne J, Brocato R (2013). A novel Sin Nombre virus DNA vaccine and its inclusion in a candidate pan-hantavirus vaccine against hantavirus pulmonary syndrome (HPS) and hemorrhagic fever with renal syndrome (HFRS). Vaccine.

