# Supplementary material for: Mechanistic basis for potent neutralization of Sin Nombre hantavirus by a human monoclonal antibody
Source: Nat Microbiol. 2023 Jun 15;8(7):1293–303. doi: 10.1038/s41564-023-01413-y (PMC10322703; doi:10.1038/s41564-023-01413-y)
Supplement: Supplementary file 1 — Supplementary Figs. 1–12 and Tables 1–3. [file 41564_2023_1413_MOESM1_ESM.pdf]

nature microbiology

Article https://doi.org/10.1038/s41564-023-01413-y

Mechanistic basis for potent neutralization of Sin Nombre hantavirus by a human monoclonal antibody

In the format provided by the authors and unedited

Table of Contents

Supplementary Figure 1. SNV-42 germline sequence alignment. 2
Supplementary Figure 2. SNV-42 escape mutations. 3
Supplementary Figure 3. Buried surface area contribution of each CDR loop. 4
Supplementary Figure 4. Sin Nombre mAb 42 escape mutants. 5
Supplementary Figure 5. Deviations from the germline encoded antibody sequence. 6
Supplementary Figure 6. Pairwise sequence alignment of the Sin Nombre virus Gn sequence with three other hantavirus species. 7
Supplementary Figure 7. A sequence alignment of Sin Nombre virus Gn^H with other hantavirus species. 8
Supplementary Figure 8. Gating strategy to detect binding of Alexa-Fluor647 labeled EC1-EC2 to SNV Gn/Gc. 10
Supplementary Figure 9. Assessing the feasibility of bivalent binding to the Sin Nombre glycoprotein lattice. 11
Supplementary Figure 10. A model of Fab SNV-42 bound to a near post-fusion conformation of the hantaviral Gn–Gc trimer. 14
Supplementary Figure 11. Fitted curves used to derive K_D values for Gn^H binding from bio-layer interoferometry experiments. 15
Supplementary Figure 12. Electron density at the interface of the SNV Gn^H:SNV-42 complex. 16
Supplementary Table 1. Biolayer interferometry kinetics for antibodies binding to SNV Gn^H. 17
Supplementary Table 2. Previously reported antibody escape mutants in the hantavirus Gn glycoprotein. 18
Supplementary Table 3: Crystallographic data collection and refinement statistics for SNV Gn-Fab 42 complex 19
Supplementary References 20

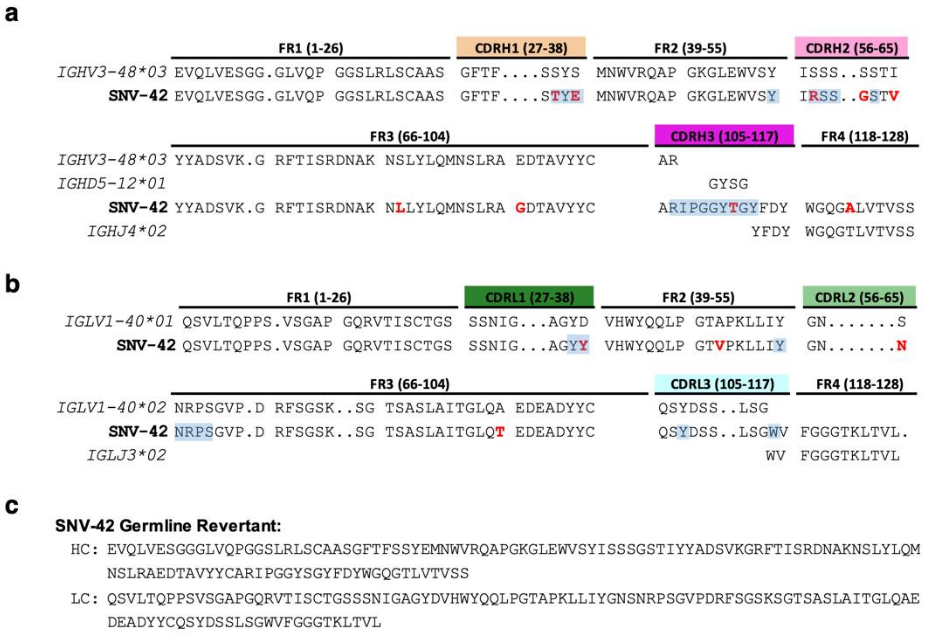

Supplementary Figure 1. SNV-42 germline sequence alignment.

Alignment of the heavy chain **(a)** and light chain **(b)** of the SNV-42 v-gene and j-gene sequences to the human germline-encoded genes. Somatically mutated residues are indicated in red. Blue highlighted residues indicate the paratope. Alignment was generated using IMGT/DomainGapAlign and IMGT numbering is used[1]. **(c)** Heavy chain (HC) and light chain (LC) sequences of the SNV-42 germline revertant.

a

Antibody	Selection virus	Replicates with escape/total replicates (%)	Mutations selected (X number of times/of Y sequenced escapes)
SNV-42	VSV/SNV	32/88 (37%)	T312K (4/6), K357Q (2/6)

b

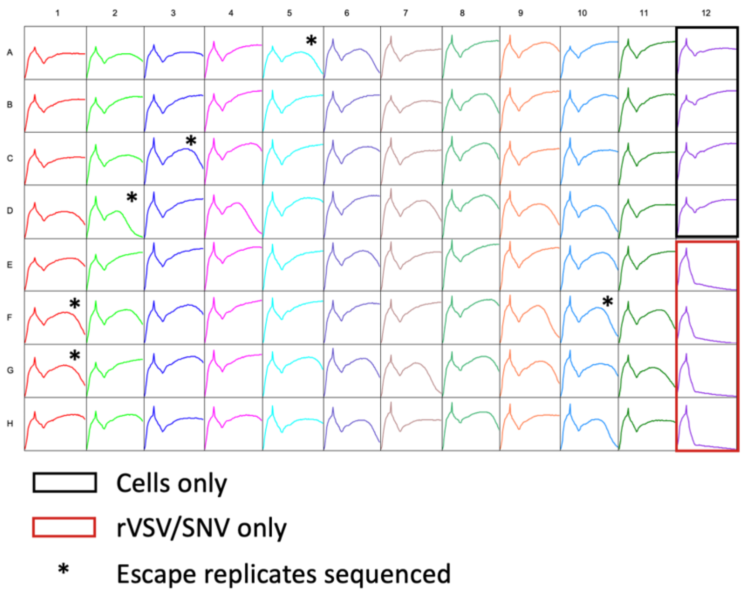

Supplementary Figure 2. SNV-42 escape mutations.

(a) Results from viral escape selection for SNV-42. Real-time cellular analysis shows the number of replicates with escape over the total number of replicates for each selection mAb against the indicated selection virus. Mutations from serial passaging were identified for each mAb, and escape was confirmed in the presence of saturating mAb concentrations.

(b) Example sensograms from individual wells of 96-well E-plate analysis showing VSV/SNV viruses that escaped neutralization by SNV-42. Control wells that contained cells only (no virus) and virus only (no SNV-42 treatment) are indicated by the black and red boxes, respectively. Wells that were sequenced (as indicated in **a)** are indicated with *.

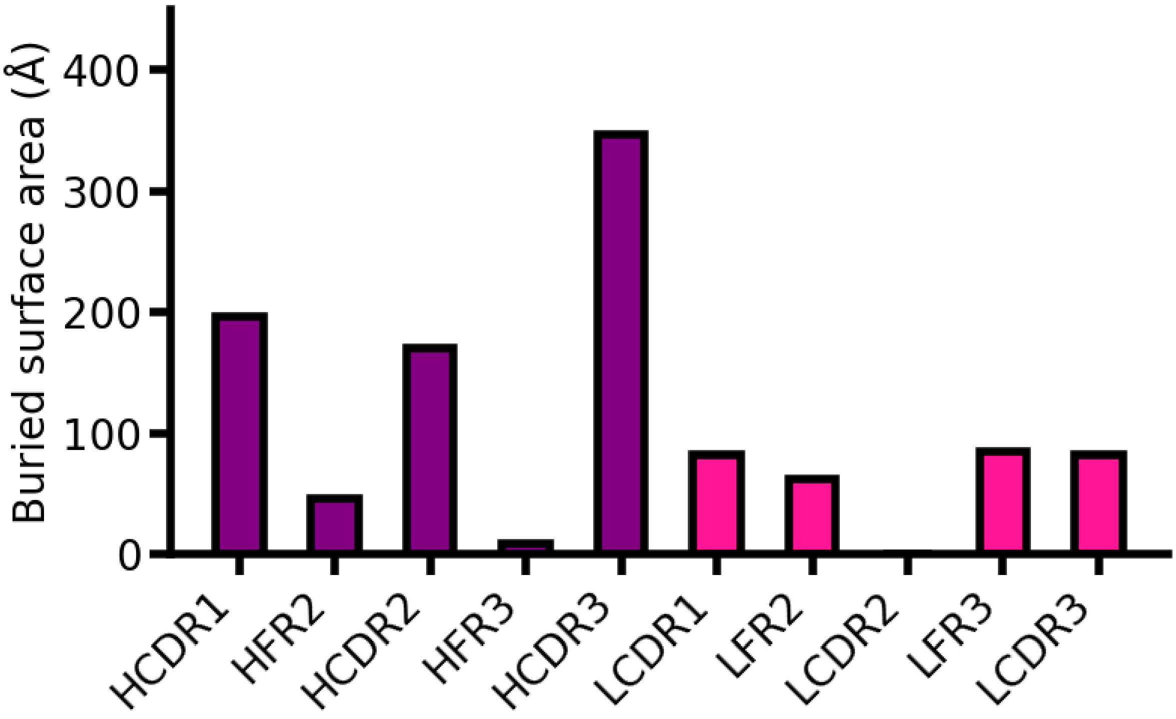

Supplementary Figure 3. Buried surface area contribution of each CDR loop.

The buried surface area between each CDR loop and the Gn was determined using the "measure buriedarea" function in ChimeraX [2].

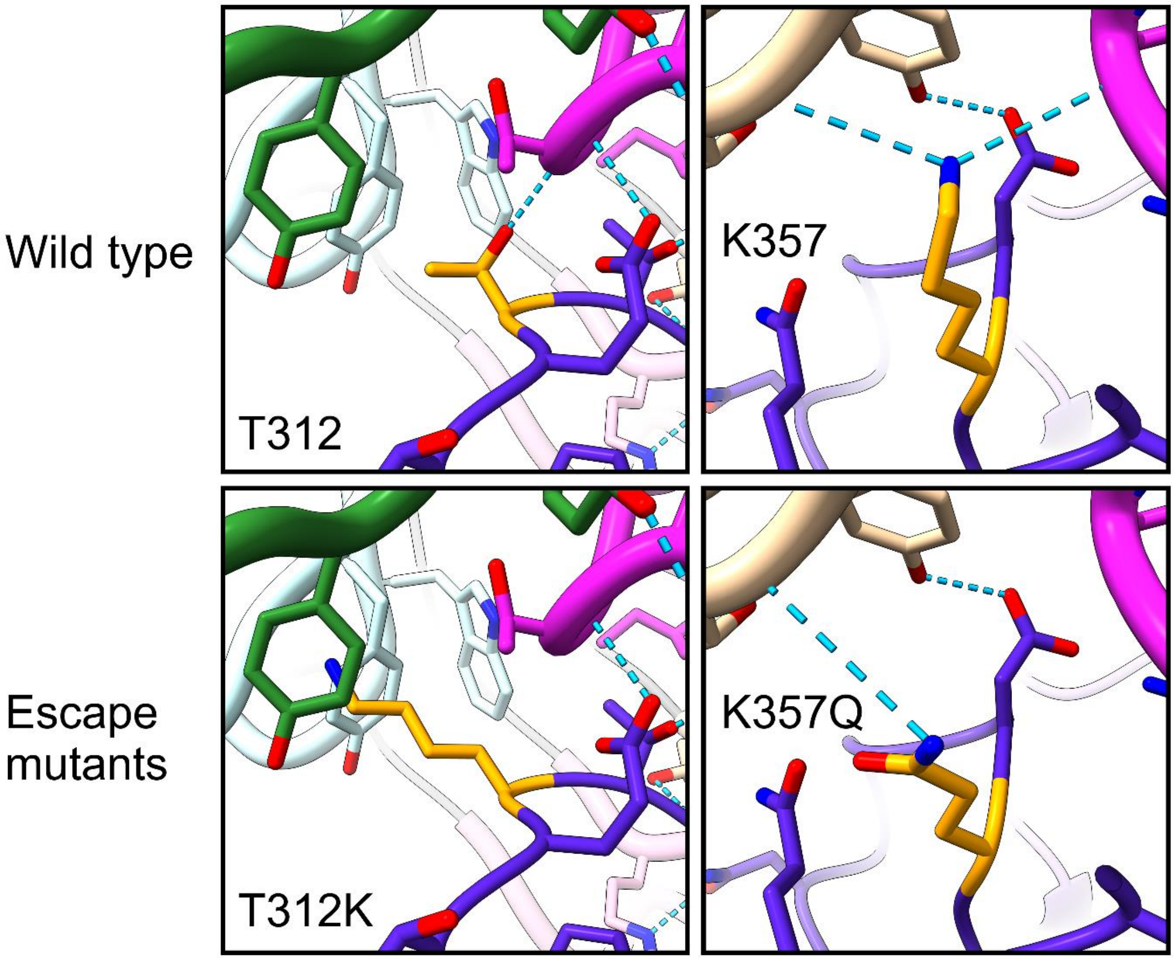

Supplementary Figure 4. Sin Nombre mAb 42 escape mutants.

Zoomed views of two escape mutants T312K and K357Q. Top, the wild-type residue as determined by X-ray crystallography. Bottom, the mutated residue is modelled as the most preferred rotamer. Hydrogen bonds are displayed as blue dashed lines, oxygen atoms in red and nitrogen atoms in blue.

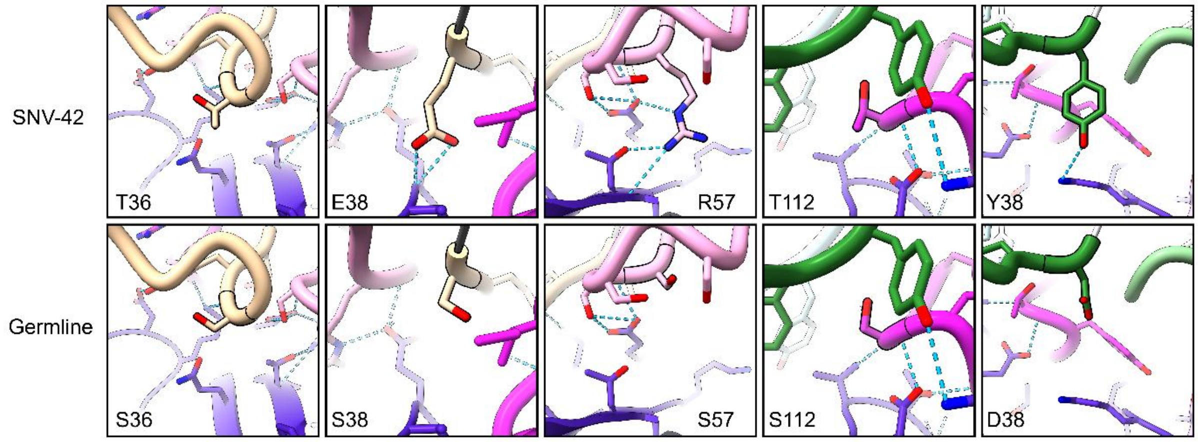

Supplementary Figure 5. Deviations from the germline encoded antibody sequence. Zoomed views of the five residues that directly interact with the Gn and are changed from the antibody germline-encoded sequence, highlighted in bold. These are T36, E38, R57 and T112 from the heavy chain and Y38 from the light chain. Top, the SNV-42 residue, as determined by X-ray crystallography. Bottom, the germline-encoded residue is modelled as the most preferred rotamer. Hydrogen bonds are displayed as blue dashed lines, oxygen atoms in red and nitrogen atoms in blue.

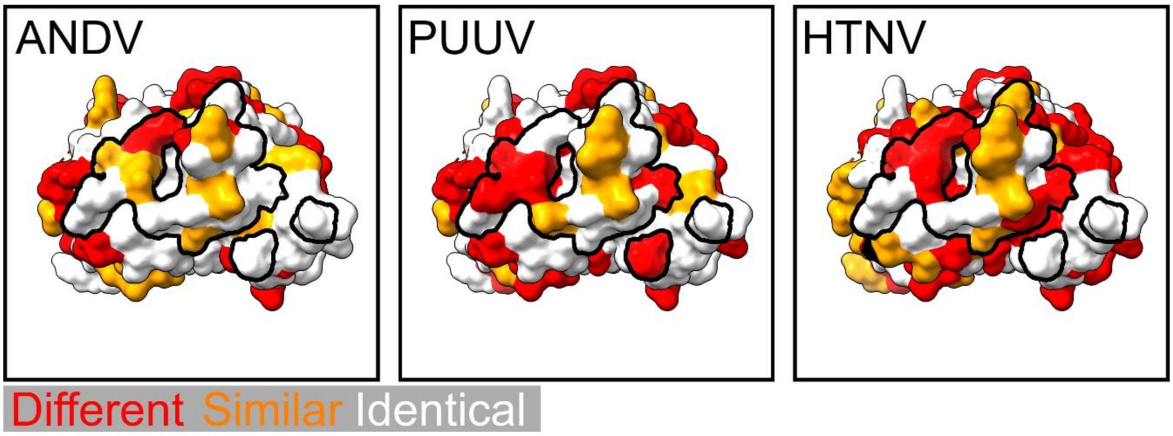

Supplementary Figure 6. Pairwise sequence alignment of the Sin Nombre virus Gn sequence with three other hantavirus species.

The following sequences were aligned using the EMBOSS Needle pairwise sequence alignment tool[3]; Sin Nombre virus (SNV, GenBank: AFV71282.1), Andes virus (ANDV, GenBank: AAO86638.1), Puumala virus (PUUV, GenBank: CCH22848.1) and Hantaan virus (HNTV, GenBank: ALI59822.1). The binding site of mAb SNV-42 is outlined in black. Identical residues are colored white, similar residues orange and non-similar residues colored red.

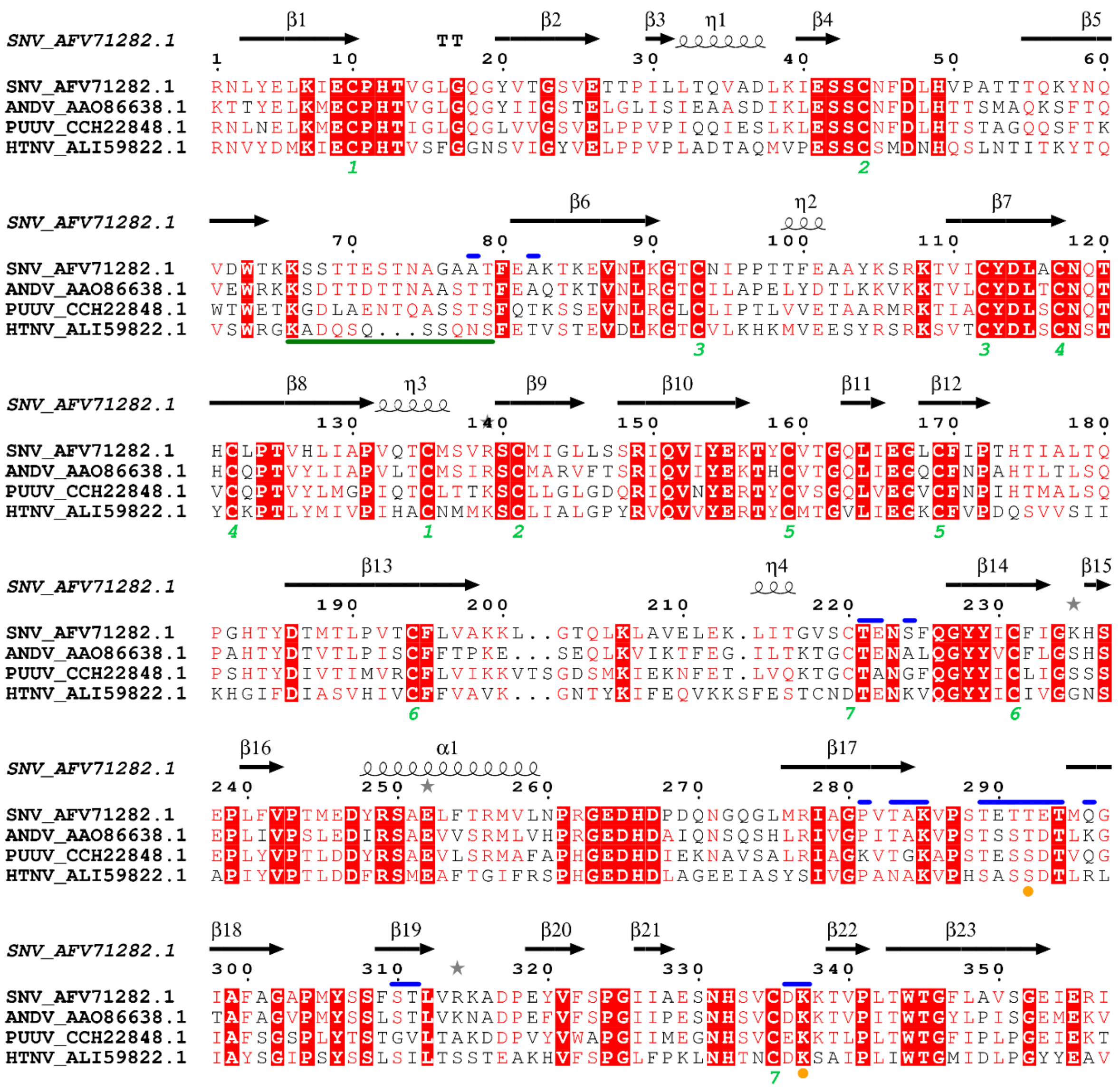

Supplementary Figure 7. A sequence alignment of Sin Nombre virus Gn^{H} with other hantavirus species.

The following sequences were aligned using Clustal Omega[4] and displayed using ESPript 3.0[5]; Sin Nombre virus (SNV, GenBank: AFV71282.1), Andes virus (ANDV, GenBank: AAO86638.1), Puumala virus (PUUV, GenBank: CCH22848.1) and Hantaan virus (HNTV, GenBank: ALI59822.1). Residues that contact mAb SNV-42 are annotated with a blue line at the top of the alignment, and the locations of two escape mutants (T312K and K357Q) are annotated with orange below the sequence. The location of the capping loop, which is

replaced by the linker sequence GGSG in the crystallized protein, is annotated with a dark green line. The serine residue in this linker forms minor contacts with the Fab. In the native SNV Gn sequence this residue corresponds to A98, as shown.

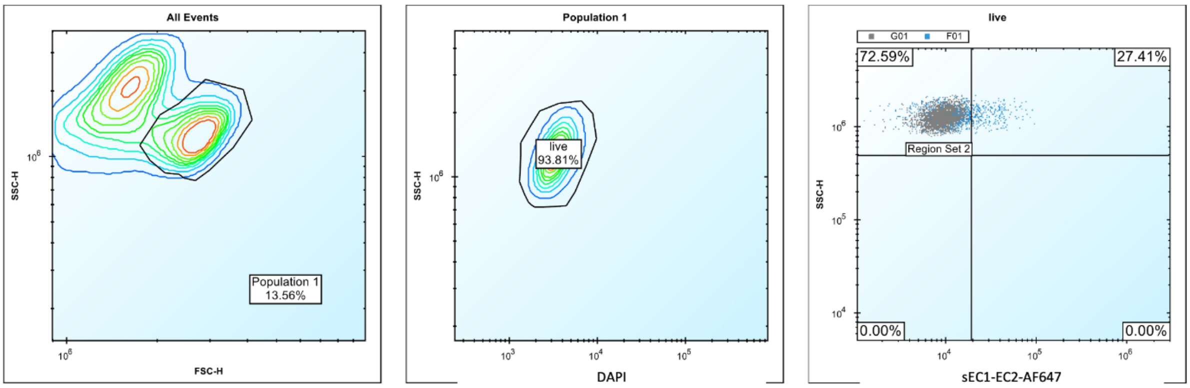

Supplementary Figure 8. Gating strategy to detect binding of Alexa-Fluor647 labeled EC1-EC2 to SNV Gn/Gc.

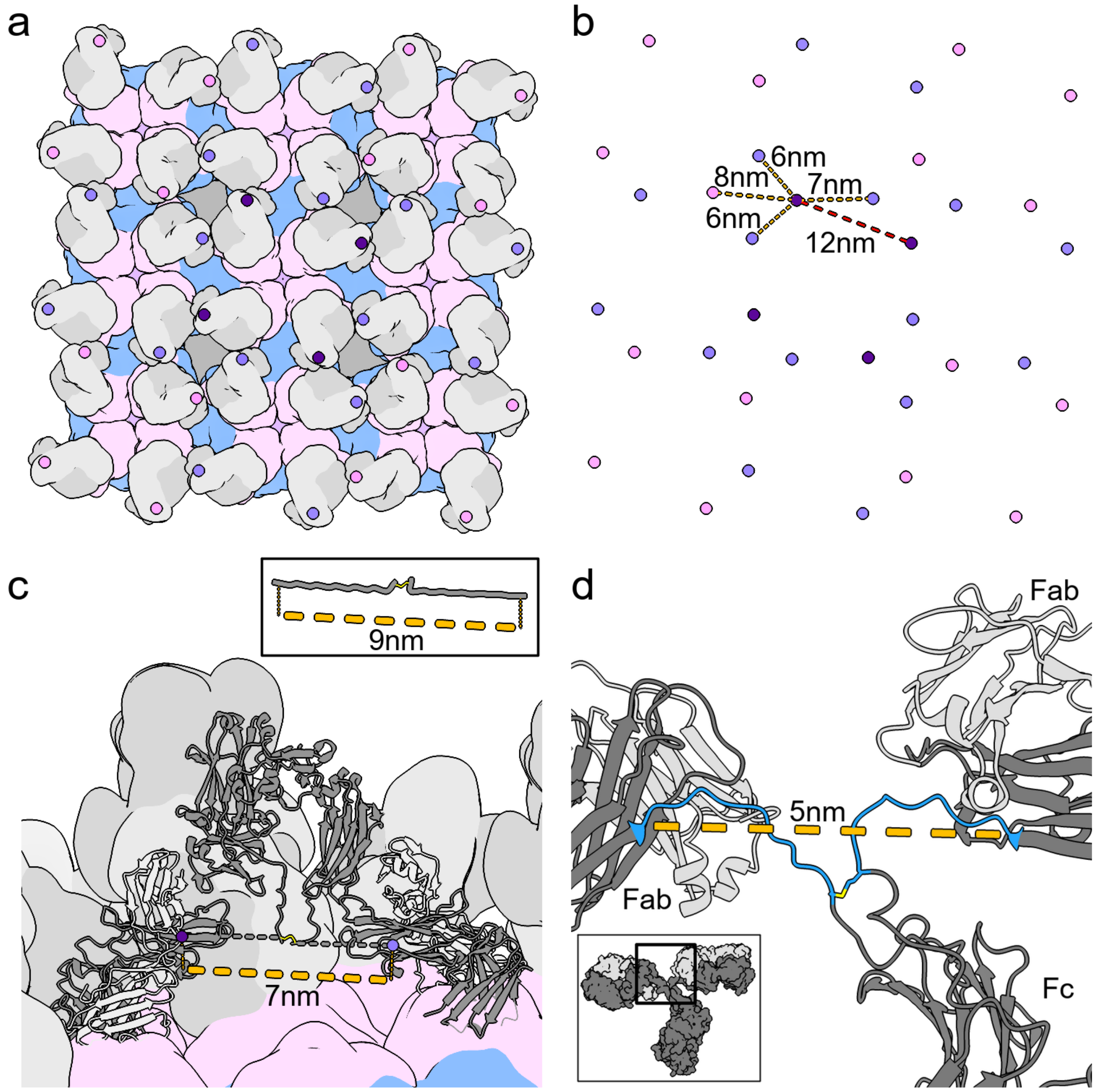

Supplementary Figure 9. Assessing the feasibility of bivalent binding to the Sin Nombre glycoprotein lattice.

a. The Sin Nombre glycoprotein lattice bound to Fab fragments of SNV-42. This assembly model is based on the previously reported ANDV glycoprotein spike tetramer (PDB: 6ZJM[6]), as displayed in **Figure 3**. The most C-terminal residue of the final Fab heavy chain β-strand was used as a common point of reference to measure

distances between Fab fragments (K223 in SNV-42). These residues are marked with circles of different colors to denote the spike to which the Fabs are bound.

b. The location of the most C-terminal residue of the final Fab heavy chain β-strand (K223 in SNV-42) from panel **a** is displayed without the underlying glycoprotein lattice for clarity. The distances between SNV-42 Fabs bound to co-localized SNV-42 epitopes on the hantavirus $(Gn\text{-}Gc)_4$ lattice are displayed. Inter-spike and intra-spike distances are colored orange and red, respectively. These distances are approximate given that significant flexibility in glycoprotein lattice and within the Fabs may be expected.

c. A zoomed view displaying a model of a single mAb spanning two SNV-42 epitopes. The location of K223 is indicated with colored circles as in panels a and b and the distance between these residues is indicated. The Fc domain displayed is taken from a previously reported full length antibody structure (PDB ID: 1HZH). Inset, a model of the missing hinge region of the mAb including K223 from each mAb heavy chain, linked by the central disulfide bond (consisting of two lengths of 13 residues). If fully extended as shown, this hinge portion could span ~9 nm (86 Å).

d. The distance spanned by one of the previously reported full-length mAb structures reported in the PDB (accession code: 1IGT). The hinge region between the most C-terminal residue of the final Fab heavy chain β-strand (equivalent to K223 in SNV-42) is colored blue. Inset a surface representation of the whole antibody indicating the region depicted. This measurement has been repeated for all the previously reported full-length (non-engineered) mAb structures that are available in the PDB (accession codes: 1HZH, 1IGY, 1IGT, 5DK3 and 6GFE), which ranges from 2–5nm (42 Å, 48 Å, 52 Å, 16 Å, and 45 Å, respectively). While the inter-spike distances of the $(Gn\text{–}Gc)_4$ lattice displayed above exceed these distances, the conformations of Fabs

within available full-length mAb crystal structures are likely limited by available crystal packing environments and therefore longer spans may be possible.

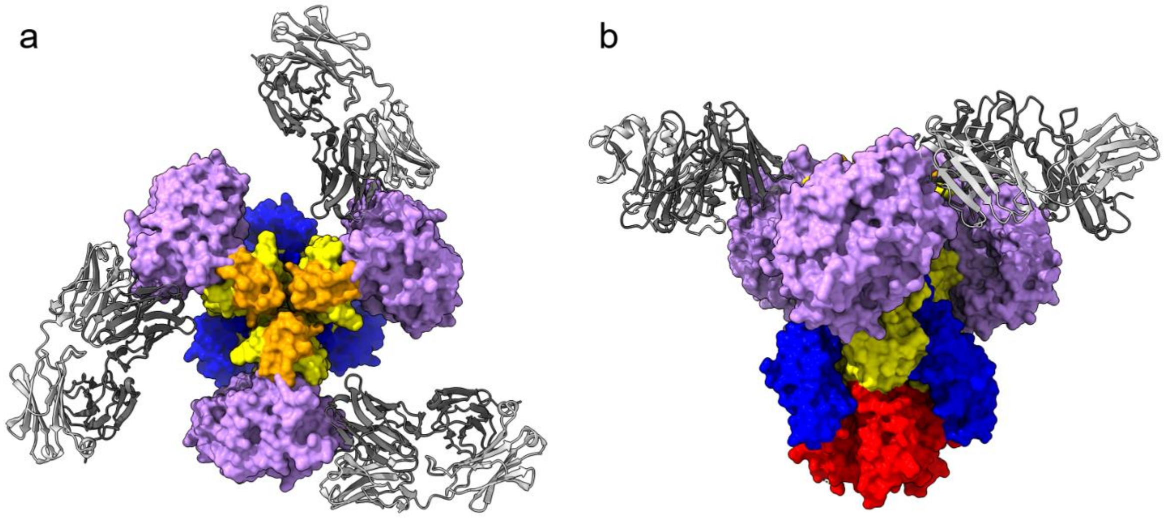

Supplementary Figure 10. A model of Fab SNV-42 bound to a near post-fusion conformation of the hantaviral Gn–Gc trimer.

A top (a) and side (b) view of a ‘near’ post-fusion Gn–Gc trimer bound to Fab SNV-42. This model is based on the previously reported ‘near’ post-fusion conformation trimer of ANDV Gn–Gc (PDB ID: 6Y5W[6]). SNV Gn was superposed onto the position of ANDV Gn. The light and heavy and light chains of Fab SNV-42 are colored dark and light grey, respectively. Gn and Gc are displayed as a surface representation and with the Gn colored pink and the domains I, II and III of the Gc colored red, yellow and blue respectively. This model demonstrates that there is no steric incompatibility with SNV-42 binding to the previously observed configuration of Gn-Gc in the ‘near’ post-fusion state[6] but does not preclude the possibility that antibody binding could interfere with the transition from pre to post fusion.

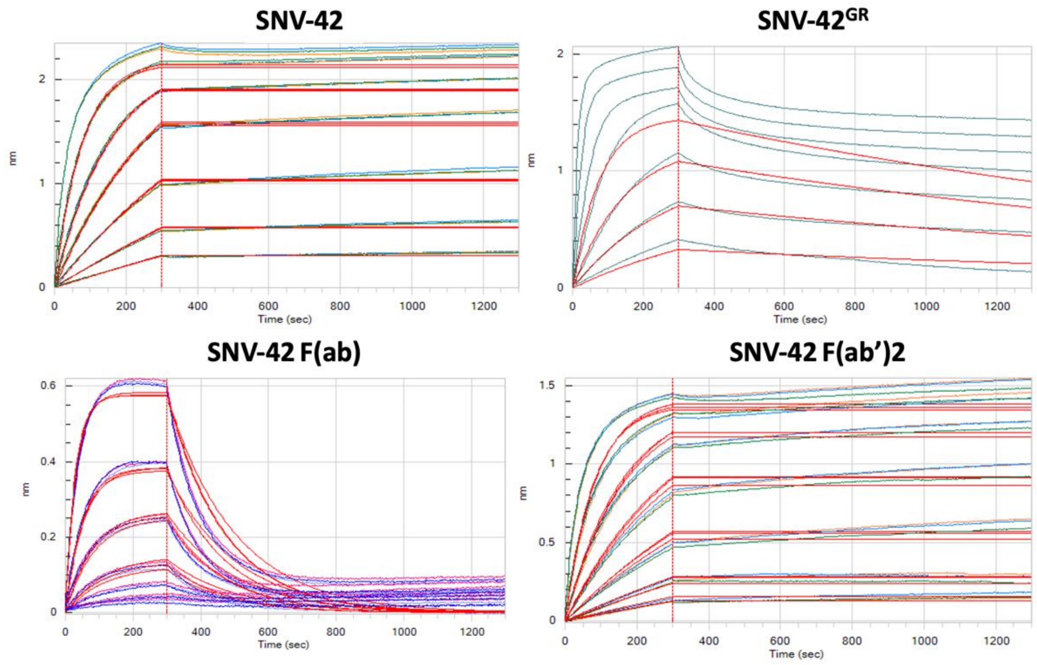

Supplementary Figure 11. Fitted curves used to derive K_D values for Gn^H binding from bio-layer interoferometry experiments.

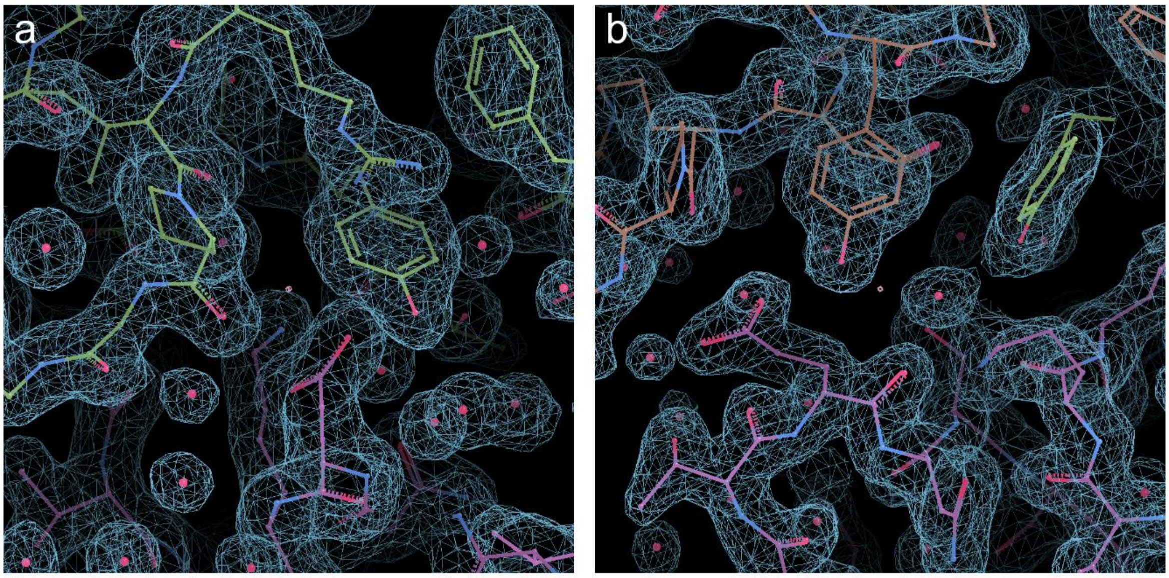

Supplementary Figure 12. Electron density at the interface of the SNV Gn^H:SNV-42 complex.

Representative views (a & b) of the maximum likelihood-weighted 2*Fo-Fc* electron density shown around the model of the SNV Gn^H:SNV-42 complex. Gn^H in purple, SNV-42 heavy chain in green and SNV-42 light chain in orange. Oxygen and nitrogen atoms in the model are displayed in pink and blue respectively. Water molecules depicted as pink dots.

Supplementary Table 1. Biolayer interferometry kinetics for antibodies binding to SNV Gn^H.

MAb	K_{on} ($M^{-1}s^{-1}$)	K_{off} (s^{-1})	K_D (M)	K_D error	X^2	R^2
**SNV-42**	1.32E+05	<1.0E-07	<1.0E-12	9.88E-12	16.4	0.99
**SNV-42^{GR}**	5.14E+05	4.54E-04	8.11E-10	1.00E-11	11.9	0.99
**SNV-42 F(ab)**	1.39E+05	5.61E-03	4.08E-08	1.15E-09	7.6	0.93
**SNV-42 F(ab′)2**	6.14E+04	<1.0E-07	<1.0E-12	<1.0E-12	12.5	0.99

Supplementary Table 2. Previously reported antibody escape mutants in the hantavirus Gn glycoprotein.

Virus	Protein	Site	mAb evaded	Homologous position in SNV Gn	Reference
HTNV	Gn	K76E	mAb 3D5 and mAb 16D2	K77	Kikuchi et al[7]
HTNV	Gn	G217R	mAb 2D5	G222	Wang et al[8]
HTNV	Gn	P303T	mAb 16E6	P307	Wang et al[8]
HTNV	Gn	H304Y	mAb 3D5	S308	Wang et al[8]
PUUV	Gn	E88D	ADI-42095	T84	Mittler et al[9]
PUUV	Gn	E95K	ADI-42093	E91	Mittler et al[9]
PUUV	Gn	Q98R	ADI-42898	N94	Mittler et al[9]
PUUV	Gn	S101N*	ADI-42898	A97	Mittler et al[9]
PUUV	Gn	D272V	mAb 5A2	E266	Horling et al[10]
ANDV	Gn	N108K	mAb KL-AN-4E1	N108	Duehr et a [11]l
ANDV	Gn	D121V	mAb KL-AN-3F6	E121	Duehr et al[11]l
ANDV	Gn	K124N	mAb KL-AN-4G11	Y124	Duehr et al[11]l
ANDV	Gn	K225R	mAb KL-AN-4H6	K226	Duehr et al[11]l

Selection of previously reported antibody escape mutants as displayed in Figure 3. * = A mutant which creates a new N-linked glycosylation site.

Supplementary Table 3: Crystallographic data collection and refinement statistics for SNV Gn-Fab 42 complex

	SNV Gn:Fab 42
**Data collection statistics**	
Beamline	DLS i04
Spacegroup	I 2 2 2
Cell dimensions	
a, b, c (Å)	88.9, 146.4, 157.7
α, β, γ (°)	90, 90, 90
Resolution range (Å)	46.6-1.80 (1.83-1.80)[a]
Wavelength (Å)	0.9795
R_{merge}	0.12 (>1.0)
I/σ I	13.4 (1.1)
$CC_{1/2}$ [a]	0.999 (0.723)
Completeness (%)	100 (99.7)
Multiplicity	13.8 (14.0)
**Refinement statistics**	
Resolution range (Å)	46.6-1.80 (1.82-1.80)[b]
No. reflections	95,137 (2,989)
R_{work} / R_{free}[c] (%)	19.0/22.1
No. atoms	
Protein	5,819
Glycan	42
Water	761
B-factors	
Protein	36.5
Glycan	54.5
Water	41.9
R.m.s deviations[d]	
Bond lengths (Å)	0.008
Bond angles (°)	1.00
Ramachandran analysis[e]	
Favored region (%)	97.4
Allowed region (%)	2.6

[a]As defined by Karplus and Diederichs[12].
[b]Numbers in parentheses refer to the outer resolution shell.
[c]R_{free} is calculated as for R_{work}, but using only 5% of the data which were separated prior to refinement.
[d]r.m.s deviations: root mean square deviation from ideal geometry.
[e]Determined using the Molprobity server[13].

Supplementary References

1. Lefranc, M.P. et al. IMGT, the international ImMunoGeneTics information system. *Nucleic Acids Res* **33**, D593-7 (2005).
2. Pettersen, E.F. et al. UCSF ChimeraX: Structure visualization for researchers, educators, and developers. *Protein Sci* **30**, 70-82 (2021).
3. Rice, P., Longden, I. & Bleasby, A. EMBOSS: the European Molecular Biology Open Software Suite. *Trends Genet* **16**, 276-7 (2000).
4. Sievers, F. et al. Fast, scalable generation of high-quality protein multiple sequence alignments using Clustal Omega. *Mol Syst Biol* **7**, 539 (2011).
5. Robert, X. & Gouet, P. Deciphering key features in protein structures with the new ENDscript server. *Nucleic Acids Res* **42**, W320-4 (2014).
6. Serris, A. et al. The Hantavirus Surface Glycoprotein Lattice and Its Fusion Control Mechanism. *Cell* **183**, 442-456 e16 (2020).
7. Kikuchi, M. et al. Characterization of neutralizing monoclonal antibody escape mutants of Hantaan virus 76118. *Arch Virol* **143**, 73-83 (1998).
8. Wang, M., Pennock, D.G., Spik, K.W. & Schmaljohn, C.S. Epitope mapping studies with neutralizing and non-neutralizing monoclonal antibodies to the G1 and G2 envelope glycoproteins of Hantaan virus. *Virology* **197**, 757-66 (1993).
9. Mittler, E. et al. Human antibody recognizing a quaternary epitope in the Puumala virus glycoprotein provides broad protection against orthohantaviruses. *Sci Transl Med* **14**, eabl5399 (2022).
10. Horling, J. & Lundkvist, A. Single amino acid substitutions in Puumala virus envelope glycoproteins G1 and G2 eliminate important neutralization epitopes. *Virus Res* **48**, 89-100 (1997).
11. Duehr, J. et al. Neutralizing Monoclonal Antibodies against the Gn and the Gc of the Andes Virus Glycoprotein Spike Complex Protect from Virus Challenge in a Preclinical Hamster Model. *mBio* **11**(2020).
12. Karplus, P.A. & Diederichs, K. Linking crystallographic model and data quality. *Science* **336**, 1030-3 (2012).
13. Chen, V.B. et al. MolProbity: all-atom structure validation for macromolecular crystallography. *Acta crystallographica. Section D, Biological crystallography* **66**, 12-21 (2010).
