# Supplementary material for: Mechanistic basis for potent neutralization of Sin Nombre hantavirus by a human monoclonal antibody
Source: Nat Microbiol. 2023 Jun 15;8(7):1293–303. doi: 10.1038/s41564-023-01413-y (PMC10322703; doi:10.1038/s41564-023-01413-y)
Supplement: Supplementary file 2 — Reporting Summary [file 41564_2023_1413_MOESM2_ESM.pdf]

nature portfolio

Corresponding author(s): James E. Crowe, Thomas A. Bowden

Last updated by author(s): Feb 22, 2023

Reporting Summary

Nature Portfolio wishes to improve the reproducibility of the work that we publish. This form provides structure for consistency and transparency in reporting. For further information on Nature Portfolio policies, see our Editorial Policies and the Editorial Policy Checklist.

Statistics

For all statistical analyses, confirm that the following items are present in the figure legend, table legend, main text, or Methods section.

n/a	Confirmed	
☐	☒	The exact sample size (n) for each experimental group/condition, given as a discrete number and unit of measurement
☐	☒	A statement on whether measurements were taken from distinct samples or whether the same sample was measured repeatedly
☐	☒	The statistical test(s) used AND whether they are one- or two-sided<br>*Only common tests should be described solely by name; describe more complex techniques in the Methods section.*
☒	☐	A description of all covariates tested
☒	☐	A description of any assumptions or corrections, such as tests of normality and adjustment for multiple comparisons
☐	☒	A full description of the statistical parameters including central tendency (e.g. means) or other basic estimates (e.g. regression coefficient) AND variation (e.g. standard deviation) or associated estimates of uncertainty (e.g. confidence intervals)
☐	☒	For null hypothesis testing, the test statistic (e.g. F, t, r) with confidence intervals, effect sizes, degrees of freedom and P value noted<br>*Give P values as exact values whenever suitable.*
☒	☐	For Bayesian analysis, information on the choice of priors and Markov chain Monte Carlo settings
☒	☐	For hierarchical and complex designs, identification of the appropriate level for tests and full reporting of outcomes
☒	☐	Estimates of effect sizes (e.g. Cohen's d, Pearson's r), indicating how they were calculated

Our web collection on statistics for biologists contains articles on many of the points above.

Software and code

Policy information about availability of computer code

Data collection	X-ray diffraction data collection; Diamond Light Source Generic Data Acquisition (GDA) software (9.28.0). Virus-infected cells expressing GFP; Immunospot BioSpot™ Analyzer (https://immunospot.com/biospot-software.html). Bio-layer interferometry; Pall FortéBio Octet Red96.
Data analysis	X-ray data scaling and reduction; xia2 (v3.8.0-g3d57088-dials-3.8), X-ray data phasing and refinement; PHENIX (v1.19.2_4158), X-ray data phasing; PHASER (v2.8.3), Protein structure model building; Coot (v0.9.4.1), Protein structure visualization; ChimeraX (v1.2.5), Sequence alignment; Clustal Omega (v1.2.4), Sequence alignment display; ESPript (v3.0), Data representation and statistical analysis; GraphPad Prism software v8 (GraphPad Prism; RRID: SCR_002798). Flow cytometry data; iQue Forecyt® Software v9.0 (https://www.sartorius.com/en/products/flow-cytometry/flow-cytometry-software). VSV escape selection and neutralization; RTCA software version 2.1.0 (Agilent/Acea Biosciences; RRID: SCR_014821). Sequence analysis of viral isolates; Geneious Prime v2020.1.2 (https://www.geneious.com/). Virus-infected cells expressing GFP; Immunospot FluoroX™ Software Suite (v7.0.18.1) (https://immunospot.com/products-filter/analyzers/fluorox-software.html). Affinity data; Analysis HT (12.2.0.2)

For manuscripts utilizing custom algorithms or software that are central to the research but not yet described in published literature, software must be made available to editors and reviewers. We strongly encourage code deposition in a community repository (e.g. GitHub). See the Nature Portfolio guidelines for submitting code & software for further information.

Data

Policy information about availability of data

All manuscripts must include a data availability statement. This statement should provide the following information, where applicable:

- Accession codes, unique identifiers, or web links for publicly available datasets
- A description of any restrictions on data availability
- For clinical datasets or third party data, please ensure that the statement adheres to our policy

Atomic coordinates and structure factors of the SNV Gn Fab SNV-42 complex have been deposited in the PDB (accession code PDB ID 8AHN). Materials used in this study will be made available but may require execution of a Materials Transfer Agreement. Source data are provided with this paper on Mendeley Data; doi: 10.17632/8fs7tgs9fs.1. The following sequences were used to design constructs for protein expression; SNV M segment, Genbank: AFV71282.1 and sEC1-EC2, GenBank: NM_002587. Two atomic models used for phasing of X-ray data were PDB ID 5OPG and PDB ID 5UR8. Additional atomic models used for data visualization were ANDV Gn/Gc spike (PDB: 6ZJM), Andes orthohantavirus Gn (PDB ID 6Y5F), Maporal orthohantavirus Gn (PDB ID 6Y62), Puumala orthohantavirus Gn (PDB ID 5FXU) and Hantaan orthohantavirus (PDB ID 5OPG).

Human research participants

Policy information about studies involving human research participants and Sex and Gender in Research.

Reporting on sex and gender	not applicable
Population characteristics	not applicable
Recruitment	not applicable
Ethics oversight	not applicable

Note that full information on the approval of the study protocol must also be provided in the manuscript.

Field-specific reporting

Please select the one below that is the best fit for your research. If you are not sure, read the appropriate sections before making your selection.

☒ Life sciences ☐ Behavioural & social sciences ☐ Ecological, evolutionary & environmental sciences

For a reference copy of the document with all sections, see nature.com/documents/nr-reporting-summary-flat.pdf

Life sciences study design

All studies must disclose on these points even when the disclosure is negative.

Sample size	Sample-size calculations were not performed to power each study. All experiments included in vitro measurements of antibody binding, receptor blocking, and virus neutralizing activities were carried out with two or more independent study replicates, which were sufficient given the large difference between activities for identified the SNV-42 antibody variants and isotype controls. FFWO, receptor binding, and mutagenesis binding studies were carried out with 6-9 techical replicates due to the high variability in the experiments, while neutralization and affinity experiments were carried out with 3 technical replicates per independent experiment due to minimal technical variable.
Data exclusions	No data were excluded from analysis.
Replication	Studies that were repeated are noted in figure captions and include all studies that demonstrated the key results reported in the manuscript. No studies have been reported that failed upon repetition. Negative control antibody, DENV 2D22, was included in all experiments to account for nonspecific activity. Consistency of mAb activity across in vitro experiments within this study and reported in the previous study (doi: 10.1016/j.celrep.2021.109086) indicate a high level of reproducibility.
Randomization	The principle of randomization is not relevant as this is an observational study.
Blinding	The investigators were not blinded for any experiments performed here. We used conventional antigen binding and virus neutralization assays using actual binding to the SNV-M antigen and VSV/SNV neutralization as the readouts. Animal studies were not conducted as part of this study.

Reporting for specific materials, systems and methods

We require information from authors about some types of materials, experimental systems and methods used in many studies. Here, indicate whether each material, system or method listed is relevant to your study. If you are not sure if a list item applies to your research, read the appropriate section before selecting a response.

Materials & experimental systems

n/a	Involved in the study	
☐	☒	Antibodies
☐	☒	Eukaryotic cell lines
☒	☐	Palaeontology and archaeology
☒	☐	Animals and other organisms
☒	☐	Clinical data
☒	☐	Dual use research of concern

Methods

n/a	Involved in the study	
☒	☐	ChIP-seq
☐	☒	Flow cytometry
☒	☐	MRI-based neuroimaging

Antibodies

Antibodies used

Previously discovered hantavirus glycoprotein-specific monoclonal antibody, SNV-42, was described in doi: 10.1016/j.celrep.2021.109086. SNV-42 was originally identified in the laboratory of James Crowe at Vanderbilt University Medical Center. SNV-42 can be provided through a Material Transfer Agreement with Vanderbilt University Medical Centre. PE conjugated goat anti-human IgG: Supplier: Southern Biotech, Catalog number: 2040-09, Clone name: polyclonal, Lot number: 0217-TH68X.

Validation

Previously discovered hantavirus glycoprotein-specific monoclonal antibodies were validated via antigen binding, virus neutralization, and in a previous paper in vivo protection studies (doi: 10.1016/j.celrep.2021.109086). All other antibodies are commercially available. Antibodies used in a specific species or application have been appropriately validated by manufacturers and this information is provided on their website and information datasheets as follows:
PE-conjugated goat anti-human-IgG (https://resources.southernbiotech.com/techbul/2040.pdf)

Eukaryotic cell lines

Policy information about cell lines and Sex and Gender in Research

Cell line source(s)

In this study we used the following cell lines: Vero (ATCC Cat# CCL-81), Expi293F (ThermoFisher Scientific, A1452), ExpiCHO (ThermoFisher Scientific, A29127), HEK293T (ECACC, 12022001).

Authentication

None of the cell lines used were authenticated.

Mycoplasma contamination

All cell lines were tested monthly and confirmed negative for mycoplasma contamination.

Commonly misidentified lines
(See ICLAC register)

None

Flow Cytometry

Plots

Confirm that:

☒ The axis labels state the marker and fluorochrome used (e.g. CD4-FITC).

☒ The axis scales are clearly visible. Include numbers along axes only for bottom left plot of group (a 'group' is an analysis of identical markers).

☒ All plots are contour plots with outliers or pseudocolor plots.

☒ A numerical value for number of cells or percentage (with statistics) is provided.

Methodology

Sample preparation

Briefly, Expi293F cells expressing either SNV Gn/Gc were washed and incubated with alexa-fluor 647 labeled extracadherin 1-2 recombinant protein for 1 h at 4°C. Cells were then stained with 4′,6-diamidino-2- phenylindole (DAPI) and subjected to flow cytometry.

Instrument

iQue Screener PLUS

Software

iQue Forecyt® Software

Cell population abundance

For a typical sample, approximately 10% of each sample fell into the "AF647+" gate, compared with <0.5% of the untransfected Expi293F population stained with the same concentration of antibody.

Gating strategy

Cells were first gated by forward and side scatter and dead cells were excluded using a viability dye (DAPI). The gate for the receptor bound subset (AF647+)was placed based on staining of untransfected Expi293F cells.

☒ Tick this box to confirm that a figure exemplifying the gating strategy is provided in the Supplementary Information.
